# Regulation of acetate tolerance by small ORF-encoded polypeptides modulating efflux pump specificity in *Methylomonas* sp. DH-1

**DOI:** 10.1186/s13068-023-02364-6

**Published:** 2023-07-18

**Authors:** Seungwoo Cha, Yong-Joon Cho, Jong Kwan Lee, Ji-Sook Hahn

**Affiliations:** 1 School of Chemical and Biological Engineering, Institute of Chemical Processes, Seoul National University, 1 Gwanak-ro, Gwanak-gu, Seoul, 08826 Republic of Korea; 2 Department of Molecular Bioscience, College of Biomedical Science, Kangwon National University, 1 Gangwondaehakgil, Chuncheon, Gangwon-do 24341 Republic of Korea

**Keywords:** Acetate tolerance, LysR-type regulator, Methanotroph, RND-type efflux pump, Small open reading frame (smORF)

## Abstract

**Background:**

Methanotrophs have emerged as promising hosts for the biological conversion of methane into value-added chemicals, including various organic acids. Understanding the mechanisms of acid tolerance is essential for improving organic acid production. WatR, a LysR-type transcriptional regulator, was initially identified as involved in lactate tolerance in a methanotrophic bacterium *Methylomonas* sp. DH-1. In this study, we investigated the role of WatR as a regulator of cellular defense against weak organic acids and identified novel target genes of WatR.

**Results:**

By conducting an investigation into the genome-wide binding targets of WatR and its role in transcriptional regulation, we identified genes encoding an RND-type efflux pump (WatABO pump) and previously unannotated small open reading frames (smORFs), *watS1* to *watS*5, as WatR target genes activated in response to acetate. The *watS1* to *watS*5 genes encode polypeptides of approximately 50 amino acids, and WatS1 to WatS4 are highly homologous with one predicted transmembrane domain. Deletion of the WatABO pump genes resulted in decreased tolerance against formate, acetate, lactate, and propionate, suggesting its role as an efflux pump for a wide range of weak organic acids. WatR repressed the basal expression of *watS* genes but activated *watS* and WatABO pump genes in response to acetate stress. Overexpression of *watS1* increased tolerance to acetate but not to other acids, only in the presence of the WatABO pump. Therefore, WatS1 may increase WatABO pump specificity toward acetate, switching the general weak acid efflux pump to an acetate-specific efflux pump for efficient cellular defense against acetate stress.

**Conclusions:**

Our study has elucidated the role of WatR as a key transcription factor in the cellular defense against weak organic acids, particularly acetate, in *Methylomonas* sp. DH-1. We identified the genes encoding WatABO efflux pump and small polypeptides (WatS1 to WatS5), as the target genes regulated by WatR for this specific function. These findings offer valuable insights into the mechanisms underlying weak acid tolerance in methanotrophic bacteria, thereby contributing to the development of bioprocesses aimed at converting methane into value-added chemicals.

**Supplementary Information:**

The online version contains supplementary material available at 10.1186/s13068-023-02364-6.

## Background

Methane is an abundant and low-cost carbon source available from natural gas and biogas. In addition, methane is a greenhouse gas with a greater effect on global warming than carbon dioxide. Therefore, there is growing interest in utilizing methane as a next-generation feedstock [1, 2]. Methanotrophic bacteria, which utilize methane as a sole carbon and energy source, are promising hosts for the biological conversion of methane into value-added chemicals. Recently, various chemicals, including lactic acid, succinic acid, indole 3-acetic acid, and cadaverine, were successfully produced through the metabolic engineering of methanotrophs [3–7]. Even though the metabolic pathways of various methanotrophs have been predicted based on genomic sequencing, transcriptome and metabolome analyses, and metabolic modeling, little is known regarding transcriptional regulatory networks. So far, only a few gene-specific transcription factors have been characterized in methanotrophs, including MmoD, involved in the regulation of methane monooxygenase (MMO) genes, and EctR1, involved in ectoine biosynthesis in *Methylomicrobium alcaliphilum* 20Z [8, 9]. However, genome-wide studies to identify target genes and functions of transcriptional regulators have not yet been reported.

Organic acids, such as lactic, succinic, 3-hydroxy propionic, itaconic, and citric acids, are widely used in the food, cosmetics, and pharmaceutical industries, also serving as building blocks for polymer production [10]. In addition, weak monocarboxylic acids, such as acetic, propionic, sorbic, and benzoic acids, are widely used as food and beverage preservatives, which inhibit microbial cell growth. Therefore, understanding the tolerance mechanisms against weak organic acids is of great relevance for the microbial production of organic acids through increasing acid tolerance and for efficient microbial control. In common, undissociated forms of organic acids in acidic medium diffuse into cells and dissociate into protons and anions in the neutral cytosol [11, 12]. Both protons and anions perturb normal cellular functions, thus inducing cellular defense mechanisms, which vary depending on the chemical structures of anions [13–15]. To improve lactate production from methane, we previously developed a lactate-tolerant strain JHM80 through adaptive laboratory evolution of *Methylomonas* sp. DH-1 [4]. We determined that overexpression of the *watR* gene (AYM39_21120/AMY39_RS21130), which encodes a LysR-type transcriptional regulator (LTTR), is partly responsible for the lactate tolerance of JHM80. LTTR is one of the largest family of bacterial regulators with diverse functions [10, 11]. LTTR has a conserved N-terminal helix-turn-helix (HTH) motif responsible for DNA binding and a C-terminal effector binding domain, which recognizes various signaling molecules regulating LTTR activity (Additional file 1: Fig. S1). The *watR* operon, consisting of the *watR* and two downstream genes, is overexpressed in JHM80 due to a 2-bp (TT) deletion in the promoter region [4]. Overexpression of the two downstream genes did not affect lactate tolerance, suggesting that overexpressed WatR may enhance lactate tolerance via the activation or repression of its target genes.

In this study, we investigated the role of WatR in regulating stress responses against weak organic acids. By investigating genome-wide binding targets of WatR and WatR-dependent transcriptional regulation, we proposed a novel role for previously unannotated small open reading frames (smORFs) in acetate tolerance.

## Results

### Differential roles of WatR in tolerance to weak organic acids

**Fig. 1:**
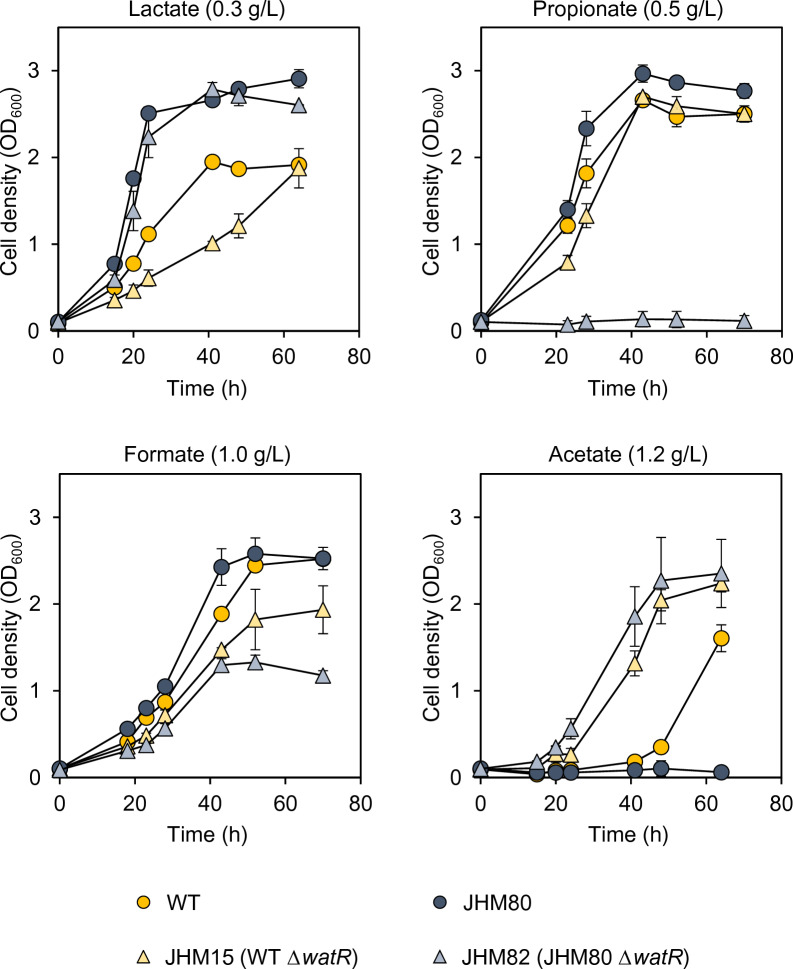
The effect of *watR* deletion in the wild-type and JHM80 strains on weak organic acid tolerance. Wild-type, JHM80, *ΔwatR* (JHM15), and JHM80 *ΔwatR* (JHM82) strains were grown in NMS media with 20% (v/v) methane containing 0.3 g/L lactate, 0.5 g/L propionate, 1.0 g/L formate, and 1.2 g/L of acetate with pH neutralization. Two independent experiments were averaged and plotted with standard deviations

We previously demonstrated that overexpression of *watR* due to a mutation in its promoter region is partly responsible for lactate tolerance in the JHM80 strain [4]. We further examined whether WatR is involved in the regulation of tolerance against other weak organic acids, including formate, acetate, and propionate, by growing cells in the presence of these acids. The mutant JHM80 strain exhibited higher tolerance against formate and propionate than its wild-type counterpart (Fig. 1). However, the tolerance phenotypes were abolished by deleting the *watR* operon (JHM82), suggesting that *watR* overexpression can enhance tolerance against propionate and formate, as well as lactate. In addition, deletion of the *watR* operon in the wild-type strain (JHM15) led to a decrease in lactate, propionate, and formate tolerance, further confirming the role for WatR in the stress response induced by these acids (Fig. 1). We previously confirmed that *watR* deletion in JHM80 reduced tolerance in the presence of 8 g/L lactate, while deletion of *watR* barely affected lactate tolerance at a lower concentration of 0.3 g/L (Fig. 1). Even without the *watR* gene, the JHM82 strain exhibited higher lactate tolerance than the wild-type strain (Fig. 1). In contrast, the JHM82 strain was more sensitive to propionate and formate than the *watR-*deleted wild-type strain (JHM15 strain, Fig. 1). These phenotypes of JHM82 strain might be due to additional mutations in the JHM80 strain conferring a selective advantage for lactate tolerance, which has not yet been characterized [4].

Intriguingly, the effect of WatR on acetate tolerance showed the opposite tendency when compared with the other acids, i.e., JHM80 exhibited lower tolerance to acetate than the wild type (Fig. 1). Deletion of *watR* in the wild type also increased acetate tolerance, suggesting that WatR may negatively affect acetate tolerance. Taken together, WatR has differential effects on tolerance depending on the type of weak organic acids: it increases tolerance against lactate, propionate, and formate while decreasing tolerance against acetate.

### Determination of genome-wide binding sites of WatR

**Fig. 2:**
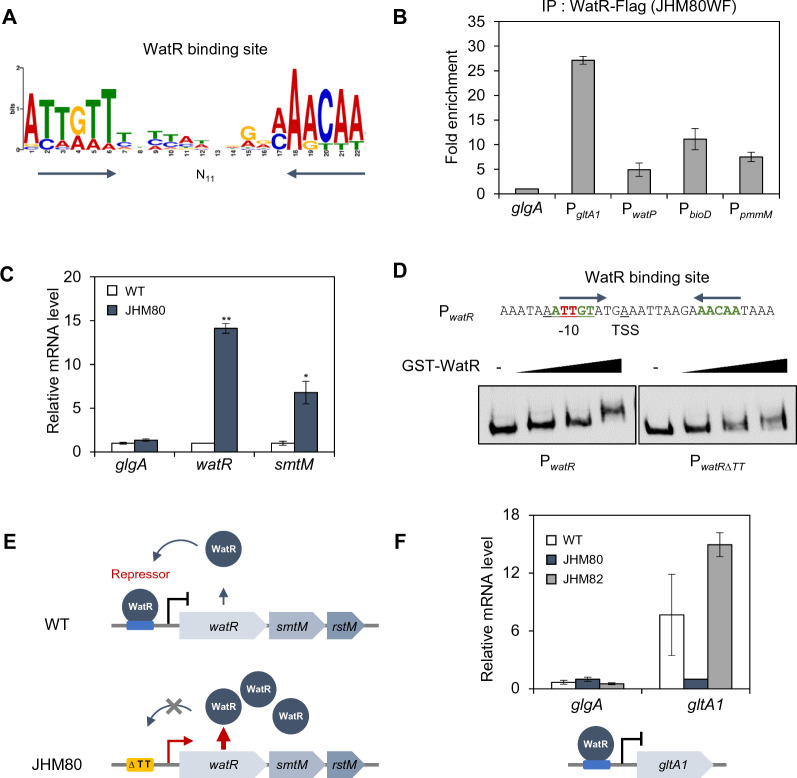
Identification of genome-wide binding sites of WatR. **A** Binding consensus sequence of WatR, discovered by ChIP-seq analysis. **B** Confirmation of WatR binding to the promoters of four selected target genes identified by ChIP-seq analysis. Binding of WatR-Flag to the target promoters in JHM80WF strain was detected by ChIP with anti-Flag antibody and indicated as fold enrichment relative to the binding to a negative control (*glgA* ORF). Each value represents the average ± standard deviations from three independent experiments. **C** Expression levels of *watR* operon genes (*watR* and *smtM*) in wild-type and JHM80 strains. The mRNA levels detected by qRT-PCR were normalized to that of *mxaF* gene and indicated as relative expression levels compared with those of wild type. The *glgA* gene was used as a negative control. Each value represents the average ± standard deviations from two independent experiments. Significant difference from wild-type strain is shown as *p < 0.1; **p < 0.05. **D** Binding of WatR to the *watR* promoter detected through in vitro EMSA assay. EMSA assay was performed by incubating GST-WatR protein with biotin-labeled *watR* promoter probes with or without TT deletion. WatR-binding sites (arrows), a putative -10 box, and transcription start site (TSS) are indicated. The deleted TT nucleotides in JHM80 are shown in red. **E** Autoregulation of *watR* expression. In wild type, WatR binds to its own promoter, repressing the expression. In the JHM80 strain, the TT deletion in the promoter prevents WatR binding, resulting in derepression of the operon. **F** Repression of *gltA1* by WatR. Expression levels of *gltA1* in the wild-type, JHM80, and JHM80 *ΔwatR* (JHM82) strains were detected by qRT-PCR. The relative mRNA levels are indicated compared with those of JHM80. Each value represents the average ± standard deviations from two independent experiments

To understand the role of WatR in weak acid tolerance, we identified genome-wide binding targets of WatR via ChIP-Seq analysis. To obtain reliable ChIP-seq signals, the *watR* gene in the genome of JHM80 strain was tagged with Flag (JHM80WF), resulting in overexpression of *watR-Flag*. The Flag tagging to WatR did not affect normal cell growth (Additional file 1: Fig. S2). We obtained 22 WatR-binding peaks, among which 16 were located in the promoter regions of annotated genes (Additional file 1: Table S5). Conserved binding motifs, ATTGTT-[N]_11_-AACAA, were identified in the WatR-binding promoter regions (Fig. 2A), which is in agreement with the palindromic binding sites (T-[N]_11_-A) previously identified for LTTRs [10]. Binding of WatR to some of the target promoters was also confirmed via ChIP-qPCR (Fig. 2B). The targets included functionally diverse genes encoding a sulfate/thiosulfate transporter and enzymes such as citrate synthase (*gltA1*), phosphomannomutase (*pmmM*), dethiobiotin synthase (*bioD*), and membrane-bound protease (*ftsH*).

### WatR functions as a repressor of its expression and *gltA1*

LTTRs are well known to autoregulate their expression [10]. As we previously reported, the expression of genes within the *watR* operon was upregulated in the JHM80 strain harboring a TT deletion in its own promoter (Fig. 2C). The deleted TT sequence is part of a putative -10 box, which overlaps with the predicted WatR-binding sequence (Fig. 2D), suggesting that the TT deletion may reduce the binding of WatR to its promoter. This idea agrees with the fact that the *watR* promoter was not detected as a WatR-binding site in our ChIP-seq experiment performed in the JHM80 strain background. Furthermore, we confirmed this hypothesis through an in vitro electrophoretic mobility shift assay with purified WatR protein (Fig. 2D, Additional file 1: Fig. S3). WatR exhibited a higher binding affinity to the wild-type *watR* promoter than the promoter harboring a TT deletion (Fig. 2D). These results suggest that WatR acts as a repressor of its expression. In the JHM80 strain, the TT deletion within the promoter region may prevent WatR binding, leading to derepression of the *watR* operon (Fig. 2E).

Among WatR target genes, *gltA1* encodes citrate synthase, which catalyzes the condensation between acetyl-CoA and oxaloacetate in the first step of the TCA cycle. Overexpression of *gltA* has been shown to eliminate acetate production and redirect carbon flux toward the TCA cycle in other bacteria [12, 13]. Therefore, we investigated whether *gltA1* expression levels are related to the WatR-dependent sensitivity to acetate stress. JHM80 exhibited lower *gltA1* expression than the wild type, which was restored following *watR* deletion, indicating that WatR represses *gltA1* (Fig. 2F). However, neither deletion nor overexpression of *gltA1* in the wild-type strain affected growth in the presence of acetate under our experimental conditions (data not shown).

### WatR activates genes encoding an efflux pump involved in general weak organic acid tolerance

**Fig. 3:**
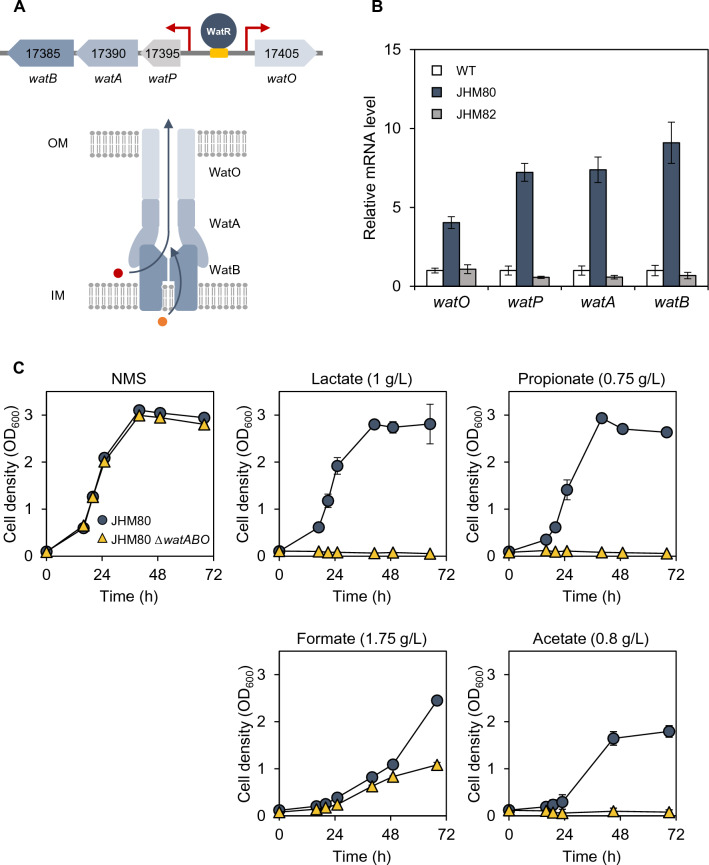
WatR-dependent activation of genes encoding an RND-type efflux pump contributing to organic acid tolerance. **A** Gene structure and putative functions of *watPAB* and *watO* genes regulated by WatR. Side view of the predicted WatABO efflux pump is shown. OM: outer membrane; IM: inner membrane. **B** WatR-dependent activation of the efflux pump genes. Transcript levels were detected by qRT-PCR in the wild-type, JHM80, and JHM80 *ΔwatR* (JHM82) strains and indicated as values relative to those of wild type. **C** The effect of deleting the efflux pump genes on acid tolerance. The JHM80 strain and JHM80 strain lacking the WatABO efflux pump (JHM87) were grown in NMS media with 20% (v/v) methane without or with the indicated weak organic acids. Each value represents the average ± standard deviations from two independent experiments

Among the WatR target genes, we identified a gene cluster encoding a resistance–nodulation–division (RND)-type efflux pump commonly found in Gram-negative bacteria. The gene cluster includes an operon consisted of the AYM39_RS17395 gene (named *watP*), 17390 (named *watA*), and 17385 (named *watB*), as well as a divergently transcribed gene, AYM39_RS17405 (named *watO*) (Fig. 3A). Although *watP* has an unknown function, *watA*, *watB*, and *watO* were predicted to encode a membrane fusion protein, inner membrane protein, and outer membrane protein, respectively, forming a tripartite complex of the RND-type efflux pump (Fig. 3A). RND pumps are known to actively transport various antibiotics, organic substances, and metals [14, 15]. Therefore, we hypothesized that this RND pump (named the WatABO pump) might be responsible for the WatR-dependent tolerance against various weak organic acids. The expression levels of these genes were higher in the JHM80 strain than in the wild type but restored when the *watR* gene was deleted in JHM80 (JHM82 strain), suggesting that overexpressed WatR activates their transcription (Fig. 3B).

Next, we focused on the role of WatABO pump in JHM80 strain which can tolerance lactate at concentrations up to 8 g/L. When the above-mentioned *watABO* genes were deleted in JHM80, it exhibited severe lactate sensitivity even in the presence of 1 g/L lactate (Fig. 3C). This result suggested that the efflux pump plays a central role in the lactate tolerance of JHM80, possibly through pumping lactate out of cells. The WatABO-deficient JHM80 strain also exhibited sensitivity toward other weak organic acids including propionate, formate, and acetate, suggesting that the efflux pump works for a wide range of weak acids (Fig. 3C). Notably, the deletion of *watR* decreased tolerance against lactate, propionate, and formate but increased acetate tolerance (Fig. 1). Therefore, although the WatR-activated efflux pump can contribute to acetate efflux, other genes regulated by WatR seem to play more dominant roles in acetate tolerance.

### Expression of WatR target genes is induced by acetate but not lactate

**Fig. 4:**
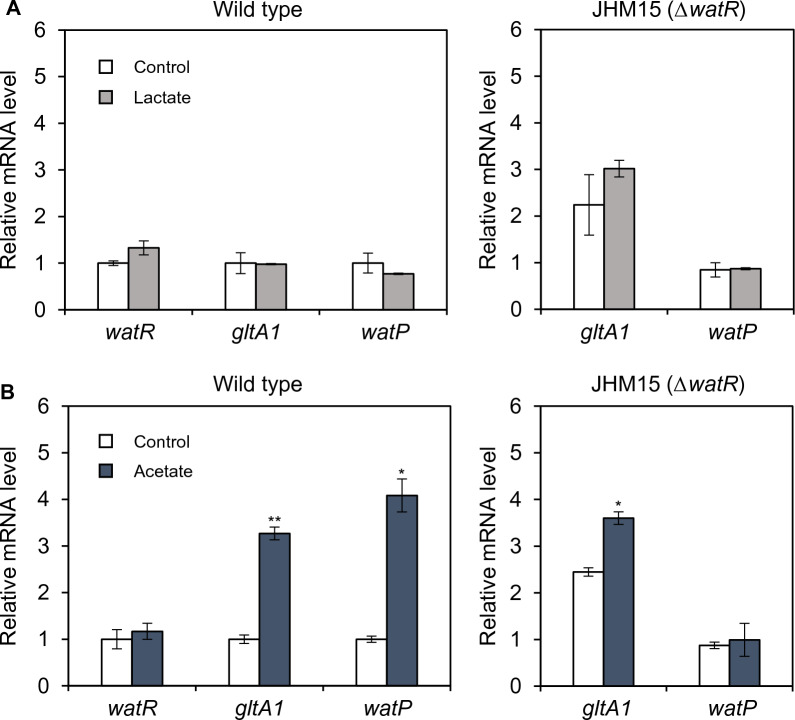
Induction of WatR target genes by acetate but not lactate. The wild-type and *ΔwatR* (JHM15) strains were grown in NMS media with 20% (v/v) methane until early exponential phase and then treated with 0.15 g/L lactate (**A**) or 0.6 g/L acetate (**B**) for 10 min. Transcript levels were detected by qRT-PCR and indicated as values relative to those of untreated wild type. Each value represents the average ± standard deviations from two (for lactate) or three (for acetate) independent experiments. Significant difference from wild-type strain is shown as *p < 0.1; **p < 0.05

Since *watR* deletion increased acetate tolerance but decreased lactate tolerance, we investigated whether the expression of *watR* target genes is regulated by these acids. Wild-type and *watR* deletion strains were treated with 0.15 g/L lactate or 0.6 g/L acetate for 10 min, which did not affect cell growth (data not shown). Although WatR overexpression increased lactate tolerance, the expression of WatR-repressed genes (*watR* and *gltA1*) and a WatR-activated gene (*watP*) was not considerably affected by lactate treatment (Fig. 4A). In contrast, acetate treatment induced the expression of *gltA*1 and *watP* (Fig. 4B). The acetate-dependent induction of *watP* and *gltA1* was diminished via *watR* deletion, suggesting that their induction mainly depended on WatR activity, regardless of whether WatR functions as an activator or repressor for basal expression (Fig. 4B). In agreement with the WatR-dependent repression of *gltA1* (Fig. 2F), the *watR* deletion mutant (JHM15 strain) exhibited higher basal expression levels of *gltA1* than the wild-type. In contrast, basal *watP* expression was not affected by *watR* deletion, suggesting that WatR activates *watP* only in the presence of acetate (Fig. 4B). However, the high basal expression level of *watP* in JHM80 suggests that high levels of WatR can activate the *watP* operon even in the absence of the inducer (Fig. 3B). The expression of *watR* gene was not induced by acetate (Fig. 4B), implying that acetate-dependent conformational changes in WatR may bring forth different effects depending on the target promoters.

### WatR regulates the expression of previously unannotated small open reading frames (smORFs)

To further understand cellular responses against acetate stress, we analyzed changes in the transcriptome following acetate treatment in both wild-type and JHM15 (*ΔwatR*) strains using RNA-seq experiments. Acetate treatment resulted in the differential expression of 72 genes by ≥ twofold (*p* < 0.05), including 49 induced genes and 23 repressed genes (Additional file 1: Table S6). The functional categories highly represented were membrane transporters in the induced genes and molecular chaperones in the repressed genes. Six of the induced genes exhibited at least twofold reduced induction in *ΔwatR,* suggesting WatR-dependent activation of these genes in response to acetate (Additional file 1: Table S6). These genes included three genes identified as direct WatR targets via ChIP-Seq analysis: AYM39_RS00605, 13560, and 17390 (*watA*). Consistent with the qRT-PCR experiments shown in Fig. 4B, the expression of *gltA1* also increased by acetate in a WatR-dependent manner. However, due to an induction fold (~ 1.8) lower than our filtration criteria, this gene was excluded from our initial selection. Two of the acetate repressed genes (AYM39_RS18690 and 18695) showed WatR-dependent repression, but WatR may regulate these genes indirectly because WatR binding to these genes was not detected (Additional file 1: Table S6).

**Fig. 5:**
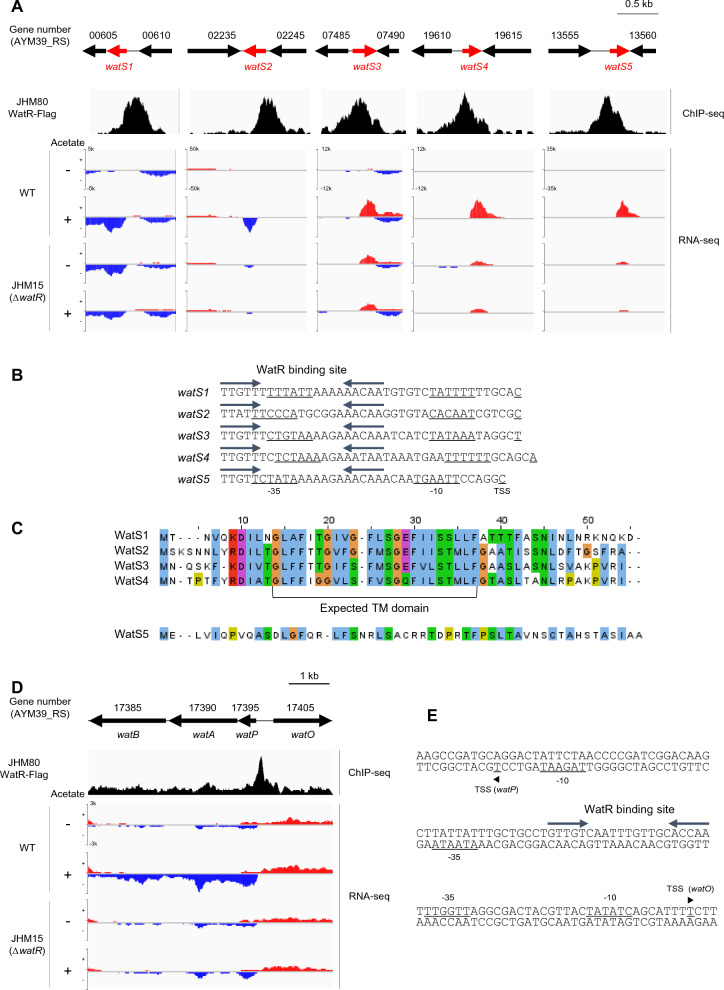
WatR-dependent regulation of smORF genes upon acetate stress. **A** WatR-dependent regulation of smORF genes. Locations of five unannotated smORFs (*watS1*-*watS5*) are aligned with the WatR-binding peaks detected via ChIP-Seq and transcript levels detected via RNA-seq using the IGV 2.3.72 program. RNA-seq analysis was performed in the wild-type and *ΔwatR* (JHM15) strains with or without acetate treatment. **B** The promoter sequences of *watS1*-*watS5* with their expected -35 box, -10 box, and TSS. The conserved WatR-binding sites are shown as inverted arrows. **C** The homology alignment of amino acid sequences of WatS1 to WatS5. A putative transmembrane domain region conserved in WatS1 to WatS4 is indicated. **D** WatR-dependent regulation of WatABO efflux pump genes. The gene locations were aligned with the WatR-binding peaks detected via ChIP-seq analysis and transcript levels detected by RNA-seq. **E** The promoter sequences of the divergently transcribed *watPAB* and *wat*O genes with their expected -35 box, -10 box, and TSS. The putative WatR-binding sites are shown as inverted arrows

Unexpectedly, when we manually analyzed the RNA-seq peaks using an integrative genomics viewer (IGV), peaks assigned as AYM39_RS00605 and 13560 genes were not mapped to these ORFs but located in the intergenic regions where we could identify short unannotated ORF containing about 50 amino acids (Fig. 5A). The expression levels of these smORFs increased upon acetate stress in the wild type but not in the JHM15 (*ΔwatR*) strain. In addition, these genes exhibited higher basal expression levels in the JHM15 (*ΔwatR*) strain. Therefore, the smORFs, named *watS1* and *watS5*, were repressed by WatR under normal conditions and activated upon acetate stress (Fig. 5A).

Based on these findings, we reexamined the 22 WatR-binding ChIP-seq peaks and RNA-seq data using the IGV browser, identifying three more WatR-regulated smORFs that had been matched with wrong ORFs or an intergenic region in our original ChIP-Seq analysis (Fig. 5A, Additional file 1: Table S5). The newly identified smORFs, named *watS2*, *watS3,* and *watS4*, also showed similar expression patterns to those of *watS1* and *watS5*, indicating the repression by WatR under normal conditions and activation upon acetate stress (Fig. 5A). In agreement with the repressor-type regulation, the well-conserved WatR-binding consensus sequences overlap with the predicted -35 box regions of these smORFs (Fig. 5B). Except for *watS5*, the polypeptides encoded by *watS1* to *watS4* showed highly homologous amino acid sequences containing a putative transmembrane domain (Fig. 5C, Additional file 1: Fig. S4).

In agreement with the qRT-PCR results shown in Fig. 4, the expression of genes encoding the RND-type efflux pump, *watPAB* and *watO*, was induced following acetate addition in the wild-type, but not the JHM15 (*ΔwatR*) strain (Fig. 5D)*.* Promoter analysis revealed that a putative WatR-binding site is located between the two expected -35 boxes of bidirectional genes (Fig. 5E).

### WatR-binding affinity to both activator and repressor target genes increased under acetate stress

**Fig. 6:**
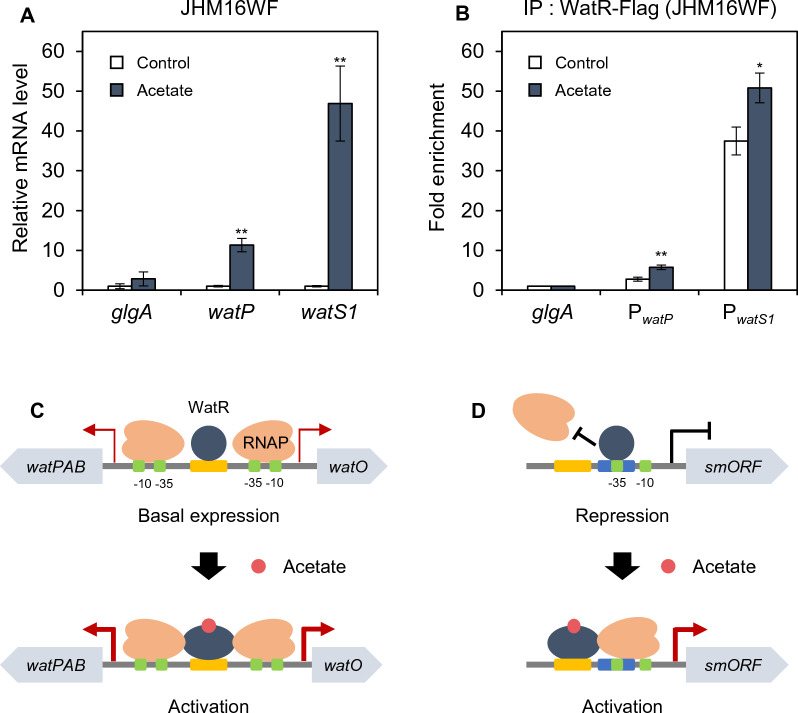
Changes in DNA binding affinity of WatR upon acetate stress. **A** Induction of *watP* and *watS1* gene expression by acetate. The JHM16WF strain expressing *watR-Flag* from the P_*EFTu*_ promoter was grown in NMS medium with 20% (v/v) methane and treated with 3.0 g/L of acetate for 10 min. The mRNA expression levels were detected by qRT-PCR and indicated as values relative to those of untreated control. Each value represents the average ± SD of the relative fold enrichment of three independent experiments, normalized to *glgA*. Significant difference from untreated sample is shown as **p < 0.05. **B** Changes in WatR DNA binding upon acetate stress. The JHM16WF strain was grown in NMS medium with 20% (v/v) methane and treated with 3.0 g/L of acetate for 10 min. ChIP analysis was performed with anti-Flag antibody and WatR binding to the promoters was detected by qPCR. Each value represents the average ± SD of the relative fold enrichment of two independent experiments, normalized to a negative control (*glgA* ORF). Significant difference from untreated sample is shown as *p < 0.1; **p < 0.05. **C** Model for the WatR-dependent transcriptional regulation of *watPAB* and *watO* genes. The WatR-binding sites does not overlap with the RNA binding sites, enabling basal transcription. Upon acetate stress, WatR activates transcription, which involves increasing DNA binding affinity. **D** Model for the WatR-dependent transcriptional regulation of smORFs. The WatR-binding sites overlap with the RNA binding sites, repressing basal transcription. Upon acetate stress, WatR activates transcription possibly by shifting the binding site to expose the RNA polymerase binding site

Next, we investigated whether the binding affinity of WatR to its target genes changes under acetate stress. Due to the relatively weak basal expression level of *watR* in the wild-type strain, immunoprecipitation experiments were not feasible. To overcome this limitation, we overexpressed *watR* using the *EFTu* (elongation factor Tu) promoter, chosen for its suitable promoter strength based on the information obtained from the promoter database [16] and our RNA-seq data. Therefore, ChIP-qPCR analysis was performed in JHM16WF strain overexpressing *watR-Flag* from the *EFTu* promoter. The WatR target genes were induced at 0.6 g/L acetate in the wild-type strain (Fig. 4B). However, due to the higher expression levels of *watR* in the JHM16WF strain, the amount of acetate had to be increased to 3.0 g/L to observe the induction of *watP* and *watS1* (Fig. 6A). We performed ChIP experiments under the same acetate stress conditions inducing the expression of *watP* and *watS1* (Fig. 6B). WatR exhibited stronger binding to the promoter of repressor-type target *watS1* than to the activator-type target *watP*. However, irrespective of the regulation type, WatR-binding affinity increased upon acetate stress (Fig. 6B).

The activity of LTTRs is typically regulated by conformational changes induced via effector binding to the C-terminal domain. Therefore, acetate itself or another metabolite generated upon acetate stress may act as a ligand regulating WatR activity. In the case of activator-type target genes, such as *watP*, the activator activity of WatR seems to be enhanced by acetate, which involves an increase in DNA binding (Fig. 6C). In the case of repressor-type target genes, such as *watS1*, WatR acts as a repressor under normal conditions (Fig. 6D). Upon acetate stress, instead of WatR derepressing target genes by being released from the promoter, WatR seems to change to an activator, possibly by shifting binding sites in the promoter, thus exposing the RNA polymerase binding site (Fig. 6D). This hypothesis is supported by the RNA-seq data showing higher acetate-induced mRNA levels of *smORF* genes in the wild-type compared to the *watR* deletion mutant (Fig. 5A).

### smORFs are responsible for acetate tolerance via efflux pump regulation

**Fig. 7:**
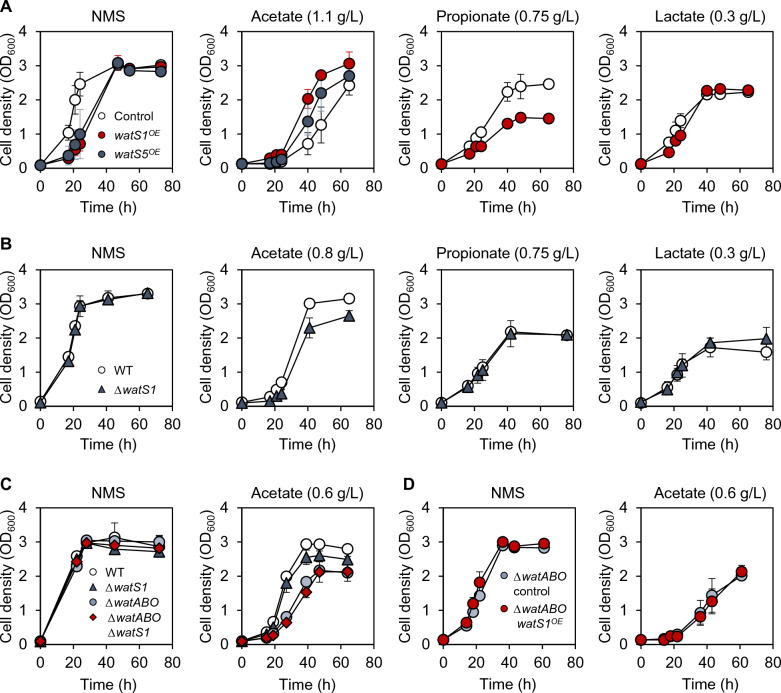
WatS1 controls acetate tolerance only in the presence of WatABO pump. **A** The effect of overexpressing the *watS1* and *watS5* genes on acid tolerance. The *ΔfliE* control strain (JHM16) and strains replacing the *fliE* gene with *watS1* or *watS5* overexpression cassette (JHM161 and JHM165) were grown in NMS media with 20% (v/v) methane without or with weak organic acids as indicated. Each value represents the average ± standard deviations from two independent experiments. **B** The effect of deleting the *watS1* gene on acid tolerance. The wild-type and *watS1* deletion (JHM17) strains were grown in NMS media with 20% (v/v) methane without or with weak organic acids as indicated. Each value represents the average ± standard deviations from two independent experiments. **C** The effect of deleting the *watS1* and WatABO pump genes. The wild-type, *ΔwatS1* (JHM17), *ΔwatABO* (JHM18), and *ΔwatABO ΔwatS1* (JHM182) strains were grown in NMS media with 20% (v/v) methane without or with 0.6 g/L acetate. Each value represents the average ± standard deviations from two independent experiments. **D** The effect of overexpressing the *watS1* gene without WatABO pump genes. The control *ΔwatABO* strain with *fliE* deletion (JHM181) and *ΔwatABO* strain overexpressing *watS1* (JHM183) were grown in NMS media with 20% (v/v) methane without or with 0.6 g/L acetate. Each value represents the average ± standard deviations from two independent experiments

Since smORFs were identified as major targets regulated by WatR upon acetate stress, we next investigated the role of these smORFs in acetate tolerance. The *watS1 and watS5* genes were overexpressed under the control of the strong *mxaF* promoter by replacing the *fliE* ORF in the genome. The *filE* site was chosen as it demonstrated high integration efficiency without negatively impacting the native physiology of the strain [4]. Overexpression of *watS1* (JHM161) and *watS5* (JHM165) increased acetate tolerance compared with the control Δ*fliE* strain (JHM16) (Fig. 7A). Furthermore, the overexpression of *watS1* showed a greater effectiveness in increasing acetate tolerance than *watS5* (Fig. 7A). Consistent with the high homology among WatS1 to WatS4 (Fig. 5C), strains overexpressing *watS2* (JHM162)*, watS3* (JHM163)*,* and *watS4* (JHM164) also showed higher acetate tolerance than that of *watS5*-overexpressing strain (Additional file 1: Fig. S5). However, overexpression of *watS1* did not improve tolerance against lactate and propionate, suggesting that WatS1 function is specific to acetate (Fig. 7A). In line with the positive effect of *watS1* overexpression on acetate tolerance, deletion of *watS1* (JHM17) decreased tolerance to acetate only but not propionate and lactate (Fig. 7B). Therefore, we further investigated acetate tolerance mechanisms induced by *watS1*.

We first confirmed that the *watS1* encodes a protein. The strain expressing the T7-tagged *watS1* (JHM161T) showed a band of the expected size (~ 7 kDa) in western blotting analysis (Additional file 1: Fig. S6). Therefore, *watS1* is expected to have a role as a smORF-encoded polypeptide (SEP). One of the known roles of SEPs is the regulation of membrane transporters. For example, AcrZ, a SEP in *E. coli*, binds to the AcrB subunit of an RND-type efflux pump, inducing conformational changes within the drug-binding pocket, which in turn affect the selectivity for transporting antibiotics [17]. Since WatR activates the expression of the WatABO pump involved in organic acid tolerance, we hypothesized that the WatR-regulated SEPs might regulate efflux pump specificity. The presence of a transmembrane domain in WatS1 to WatS4 also supports our hypothesis. The JHM18 strain with deletion of the efflux pump genes (*ΔwatABO*: *ΔwatPAB* and *ΔwatO*) exhibited higher acetate sensitivity than the *ΔwatS1* strain (Fig. 7C). However, additional deletion of *watS1* in the efflux pump deletion mutant (JHM182) did not further increase acetate sensitivity (Fig. 7C), suggesting that WatS1 and the WatABO pump might work in the same pathway. In addition, overexpression of the *watS1* gene increased acetate tolerance in the wild type (Fig. 7A) but could not rescue the acetate sensitivity of the *ΔwatABO* control strain (JHM181) (Fig. 7D), further supporting the hypothesis that WatS1 might function through the WatABO pump.

**Fig. 8:**
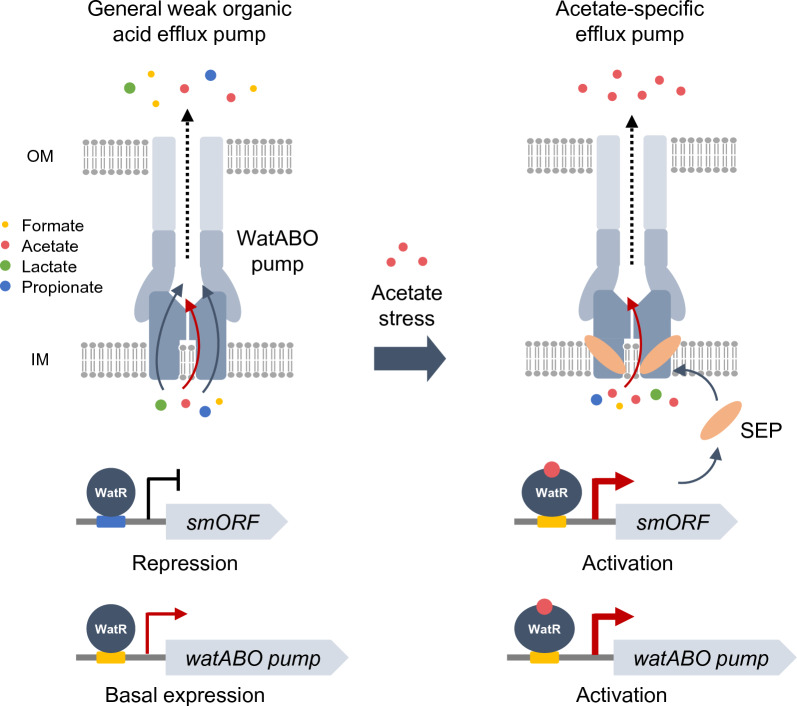
Model for SEP-dependent regulation of the WatABO pump upon acetate stress. Under normal conditions, the WatABO pump extrude a wide range of weak organic acids including acetate, formate, lactate, and propionate. Upon acetate stress, activated WatR induces transcription of WatABO pump and *watS* smORF genes. WatS SEP binds to the WatABO pump, increasing the specificity toward acetate for efficient removal of acetate from cells

Taken together, we propose a working model of WatS1-4 controlling the specificity of the WatABO efflux pump (Fig. 8). The efflux pump functions as a general transporter for several weak organic acids, including formate, acetate, lactate, and propionate under normal conditions. In response to acetate stress, the expression of the efflux pump genes and *watS1-4* is activated in a WatR-dependent manner. The SEPs WatS1-4 may then interact with the WatABO pump, shifting specificity toward acetate. This regulatory mechanism enables efficient cellular protection against acetate by switching the general weak organic acid efflux pump to an acetate-specific efflux pump in the presence of acetate.

## Discussion

### Regulation of WatR activity under acetate stress

Understanding the acid tolerance mechanisms is critical for improving microbial production of useful organic acids. There is growing interest in utilizing methanotrophs for the bioconversion of methane into value-added chemicals, but little is known about their acid stress responses. In this study, we elucidated the role of WatR, an LTTR, as a regulator of weak organic acid stress responses in *Methylomonas* sp. DH-1. Through the analysis of genome-wide binding targets of WatR and WatR-dependent transcriptional regulation, we identified that WatR functions both as a transcriptional repressor and an activator, with its activity regulated by acetate.

LTTR is among the largest families of bacterial regulators with diverse functions including stress response, biosynthesis, and biodegradation in response to various effector molecules binding to the C-terminal domain [10]. In agreement with the classical regulatory model of LTTRs, WatR autoregulates its expression and activates target gene expression in response to acetate. WatR represses the basal expression of certain target genes, such as *watR*, *gltA*, and smORFs (*watS1* ~ *watS*5). Within their promoters, the WatR-binding site overlaps with the RNA polymerase binding site; so, access of the RNA polymerase is inhibited via WatR binding. In contrast, WatR overexpression activates the basal transcription of divergently transcribed genes encoding an RND-type efflux pump (*watPAB* operon and *watO*), where the WatR-binding site does not overlap with the RNA polymerase binding site. In both cases, acetate treatment increased WatR binding to the target promoters and increased transcription. Therefore, conformational changes in WatR upon acetate stress may convert it to a transcriptional activator. In the case of WatR-repressed genes, rather than being derepressed via release of WatR from the promoter, acetate-dependent activation may shift the WatR-binding site wherein WatR can activate transcription instead of preventing RNA polymerase binding. Such an effector-dependent transition from a repressor to an activator through changes in the binding sites has also been reported in other LTTRs [18]. Acetate is known to directly regulate AlsR, an LTTR modulating acetoin production in *Bacillus subtilis* [19]. In *Saccharomyces cerevisiae*, weak organic acids, including acetate, can directly bind to the Haa1 and War1 transcription factors involved in the cellular response against weak organic acids [20]. Therefore, acetate may serve as a direct effector for WatR, but further studies are necessary to identify the specific effector molecules for WatR regulation.

### Roles of WatS SEPs as regulators of increasing acetate specificity of the WatABO efflux pump

Cells have evolved defense mechanisms against weak acids generated during normal cell growth or present in the environment. The major cellular defense mechanisms against weak acid stress include the export of excess cytosolic protons and acid anions through membrane transporters, restricted diffusion of weak acids by remodeling the cell wall and plasma membrane, and the metabolic conversion of weak acids [21–23]. In this study, we demonstrate that WatR can affect tolerance against a wide range of weak organic acids, having a more specific role in response to acetate stress. We propose a novel defense mechanism against acetate stress: the SEP-mediated regulation of efflux pump specificity.

The WatR-activated WatABO pump was identified to work as an efflux pump for general weak organic acids, including formate, acetate, lactate, and propionate. Upon acetate stress, WatS SEPs may interact with the WatABO pump, changing its specificity toward acetate, leading to more efficient removal of acetate out of cells. The expression of *watS1* to *watS5* is repressed by WatR under normal conditions and activated only upon acetate stress. In contrast, cells express basal or upregulated WatABO pump genes under normal and acetate conditions, respectively. This hypothesis is based on the well-established example of AcrB regulation by AcrZ, an SEP, in *E. coli*. AcrB is an inner membrane-binding component of an RND-type efflux pump exporting several antibiotics, organic solvents, and detergents [24–26]. Genetic and cryo-EM-based structural studies revealed that AcrZ binding to AcrB leads to conformational changes in the drug-binding pocket of AcrB, altering specificity toward certain antibiotics, such as chloramphenicol [17, 24]. In agreement with this working model, overexpression of *watS* smORFs increased tolerance against acetate, but not other weak acids, only in the presence of the WatABO pump. We tried to detect a direct interaction between the WatS1 polypeptide and WatB inner membrane subunit through co-immunoprecipitation and split GFP assay. However, we were unsuccessful due to technical difficulties in tagging WatB without affecting cell growth.

The proposed role of WatR is in cellular defense against acetate stress, which contradicts the observed acetate-resistant phenotype of the *watR* deletion mutant. This inconsistency might be related to our experimental conditions of acetate stress. Since acetate was added at the beginning of the culture, the basal expression levels of defense genes might affect the tolerance phenotypes we observed. Although WatR-dependent gene expression may play an important role in the cellular adaptation to dynamic changes in acetate levels, the high basal expression of *watS* genes and other WatR-repressed genes in the inoculum may be beneficial for survival of the *ΔwatR* strain under our acid stress conditions. As the *watR* deletion decreased tolerance against formate, lactate, and propionate, tolerance against these weak acids seems more dependent on WatR target genes, such as WatABO pump genes, which are activated but not repressed by WatR. This requires further studies to understand the role of other WatR target genes in tolerance against different weak organic acids.

The identified role of WatR is similar to that of YdcI, an LTTR found in a wide range of Gram-negative bacteria [27]. Although WatR and YdcI have low sequence homology, YdcI is known to be involved in the acid stress response and pH homeostasis in *Salmonella enterica* serovar Typhimurium and *E. coli* [28, 29]. In addition, both WatR and *E. coli* YdcI repress the expression of citrate synthase, and deletion of *watR* and *ydcI* both increased acetate tolerance [27]. Although we could not observe the contribution of the citrate synthase gene (*gltA1*) in acetate tolerance under our experimental conditions, we cannot rule out the possibility that the WatR-dependent activation of *gltA1* may contribute to alleviating acetate stress through the upregulation of acetate flux toward to the TCA cycle.

### Emerging roles of smORFs in stress responses

Comprehensive analysis of the ChIP-seq and RNA-seq data revealed five unannotated smORFs, *watS1* to *watS5*, as WatR target genes repressed under normal conditions and activated upon acetate stress. The smORFs are usually ignored in gene annotation programs, which use cut-off sizes of 50 and 100 amino acids for prokaryotes and eukaryotes, respectively [30]. However, recent advances in genomics, proteomics, and bioinformatics have enabled the discovery of previously unannotated smORFs from bacteria to humans [31–35]. smORFs and SEPs fall into two main functional categories: SEPs with their own function and upstream ORFs (uORFs) regulating the translation of a downstream gene in eukaryotes. In both cases, growing evidence supports the prevailing role of smORFs in stress responses. Translation of eukaryotic uORF prevents scanning and/or re-initiation at the downstream ORF, which can regulate stress-dependent translation of the downstream gene [36]. In bacteria, the expression of smORFs is induced under various stress conditions, including heat shock, cold shock, oxidative stress, low pH, and different nutrient conditions [37–39]. To date, only a few functional SEPs have been characterized, but they commonly regulate biological functions by modulating the activity or stability of other proteins or protein complexes [40]. In addition, various functional SEPs have been identified as membrane proteins. Recent metagenomic analysis of the human microbiome revealed approximately 4000 SEPs, about 30% of which are predicted to be secreted or membrane-bound [41]. Like the proposed role of WatS in regulating the WatABO pump, several SEPs with one transmembrane domain are known to regulate membrane transporters in response to environmental signals, including nutrients and metal ions [42, 43]. The SEP-dependent transporter regulation has also been reported in mammals. DWORF, an SEP localized within the sarcoplasmic reticulum membrane, interacts with the Ca^2+^-ATPase SERCA, thus increasing Ca^2+^ uptake [44].

Although the RND-type efflux pump is well known for transporting a broad range of chemicals, the SEP-dependent regulation of substrate specificity may provide an efficient and rapid cellular adaptation in response to environmental stress. The *watP* gene of unknown function in the *watPAB* operon is also predicted to encode a relatively short protein of 89 amino acids, which has two transmembrane domains. Therefore, WatP might also act as a regulator or subunit of the WatABO pump. Our study highlights the important roles of SEPs in fine-tuning stress responses by modulating specific interacting proteins. The *Methylomonas* sp. DH-1 genome contains at least 10 RND pump genes. Our research is focused on acetate-responsive smORFs, but it would be interesting to determine whether SEPs regulate other RND pumps or transporters in response to different stress conditions. Understanding the SEP-dependent efflux mechanisms of various weak organic acids can contribute to the production of diverse organic acids in methanotrophs and other bacterial hosts that may share the same regulatory strategy of organic acid efflux.

## Conclusions

In this study, we have investigated the role of WatR transcription factor in regulating cellular defense against weak organic acids, particularly focused on its response to acetate stress. By investigating genome-wide binding targets of WatR and WatR-dependent transcriptional regulation, we have identified previously unannotated smORFs and the genes encoding the WatABO efflux pump as WatR target genes activated in response to acetate. Our findings suggest that these short polypeptides encoded by smORFs may enhance the specificity of the WatABO pump toward acetate, thereby switching general weak acid efflux pump to an acetate-specific efflux pump for efficient cellular defense against acetate stress.

## Methods

### Strains and culture conditions

All strains used in this study are listed in Table 1. Strains derived from *Methylomonas* sp. DH-1 (KCTC13004BP) were cultured in 3 mL nitrate mineral salts (NMS) medium (0.49 g/L MgSO_4_, 1.0 g/L KNO_3_, 0.23 g/L CaCl_2_·2H_2_O, 3.8 mg/L Fe-EDTA, 0.5 mg/L Na_2_MoO_4_, 10 μM CuSO_4_·5H_2_O, with the addition of trace element solution, vitamin stock and phosphate stock solution: recipes of these solutions are in Additional file 1: Table S1) with 20% (v/v) methane in a 30 mL serum bottle capped with a butyl rubber stopper at 30 °C with shaking at 170 rpm [4]. For chromatin immunoprecipitation (ChIP) and ChIP-seq experiments, strains were cultured in 50 mL NMS medium with 20% (v/v) methane in a 500 mL baffled flask sealed with rubber type screw cap.

**Table 1:** Strains used in this study

Strain	Description	Genotype	References
*Methylomonas* sp. DH-1	Wild-type strain		[54]
JHM15	*watR* operon deletion in DH-1	DH-1 *Δ(watR-smtM-rstM)::Kan*^*R*^	This study
JHM16	*fliE* deletion in DH-1	DH-1 *ΔfliE::kan*^*R*^	This study
JHM161	*watS1* overexpression in DH-1	DH-1 ΔfliE:: P_*mxaF*_-watS1-T_*rrnB*_*-Kan*^*R*^	This study
JHM162	*watS2* overexpression in DH-1	DH-1 *ΔfliE::* P_*mxaF*_-*watS2-*T_*rrnB*_*-Kan*^*R*^	This study
JHM163	*watS3* overexpression in DH-1	DH-1 *ΔfliE::* P_*mxaF*_-*watS3-*T_*rrnB*_*-Kan*^*R*^	This study
JHM164	*watS4* overexpression in DH-1	DH-1 *ΔfliE::* P_*mxaF*_-*watS4-*T_*rrnB*_*-Kan*^*R*^	This study
JHM165	*watS5* overexpression in DH-1	DH-1 *ΔfliE::* P_*mxaF*_-*watS5-*T_*rrnB*_*-Kan*^*R*^	This study
JHM17	*watS1* deletion in DH-1	DH-1 *ΔwatS1::Kan*^*R*^	This study
JHM18	WatABO pump deletion in DH-1	DH-1 *Δ(watPAB-watO)::Amp*^*R*^	This study
JHM181	*fliE* deletion in JHM18	JHM18 *ΔfliE::Kan*^*R*^	This study
JHM182	*watS1* deletion in JHM18	JHM18 *ΔwatS1::Kan*^*R*^	This study
JHM183	*watS1* overexpression in JHM18	JHM18 Δ*fliE*::P_*mxaF*_-*watS1-*T_*rrnB*_*-Kan*^*R*^	This study
JHM80	Evolved strain from DH-1		[4]
JHM82	*watR* operon deletion in JHM80	JHM80 *Δ(watR-smtM-rstM)::Kan*^*R*^	[4]
JHM87	WatABO pump deletion in JHM80	JHM80 *Δ*(*watPAB-watO*)*::Kan*^*R*^	This study
JHM16WF	*watR-Flag* overexpression in DH-1	DH-1 Δ*fliE*::P_*EFTu*_-*watR*-Flag-T_*rrnB*_*-Kan*^*R*^	This study
JHM80WF	*watR-Flag* tagging in JHM80	JHM80 *watR*-*Flag*-T_*rrnB*_*-Kan*^*R*^	This study
JHM161T	*watS1*-T7 overexpression in DH-1	DH-1 Δ*fliE*::P_*mxaF*_-*watS1-* T7-T_*rrnB*_*-Kan*^*R*^	This study

### Plasmid construction

Plasmids and primers used in this study are listed in Table 2 and Additional file 1: Table S2. Plasmids for deletion were generated based on the pDel2-fliE plasmid by replacing the chromosome targeting sequences for *fliE* with 1-kb upstream and downstream sequences of the target genes. To generate pDel-watABPO(A) plasmid, ampicillin resistance gene (*Amp*^*R*^) was PCR amplified from pCM184 [45] and cloned between ApaI and PacI site, replacing  the kanamycin resistance gene (*Kan*^*R*^) of Del2-watABPO(K). For DNA integration via substituting *fliE*, plasmid pFliE-mxaF containing [U_*fliE*_-T_*rrnB*_-P_*mxaF*_-T_*rrnB*_-*Kan*^*R*^-D_*fliE*_] cassette was generated by inserting P_*mxaF*_ promoter using MauBI and BamHI sites, and T_*rrnB*_ terminator using AscI and MauBI sites between the U_*fliE*_-T_*rrnB*_ cassette of pDel2-fliE plasmid. T_*rrnB*_ terminator was inserted right after the U_*fliE*_ cassette to prevent transcription from *fliE* promoter after genome integration. The genes of interest were cloned between the promoter and terminator using BamHI and SpeI sites for overexpression. To make pFliE-EFTu-watR-Flag plasmid, P_*mxaF*_ of pFliE-mxaF was substituted to P_*EFTu*_ by MauBI and BamHI, and Flag tag containing *watR* ORF was cloned with BamHI and *Spe*I. pWatR-G4S-Flag was designed to insert the Flag tag sequence with G4S linker before the stop codon of the *watR* ORF [46]. The upstream homology region was amplified with reverse primer containing G4S linker, Flag tag sequence, and stop codon, and then cloned into pDel2 using *Not*I and *Spe*I.

**Table 2:** Plasmids used in this study

Plasmid	Description	References
Plasmids for gene deletion in *Methylomonas* sp. DH-1		
pDel2-WSR	pDel2-U_*watR*_-[T_*rrnB*_-*Kan*^*R*^]-D_*rstM*_	[4]
pDel2-fliE	pDel2-U_*fliE*_-[T_*rrnB*_-*Kan*^*R*^]-D_*fliE*_	[4]
pDel2-watABPO(A)	pDel2-D_*watO*_-[T_*rrnB*_-*Amp*^*R*^]-D_*watA*_	This study
pDel2-watABPO(K)	pDel2-D_*watO*_-[T_*rrnB*_-*Kan*^*R*^]-D_*watA*_	This study
pDel2-watS1	pDel2-U_*watS1*_-[T_*rrnB*_-*Kan*^*R*^]-D_*watS1*_	This study
Plasmids for gene expression in *Methylomonas* sp. DH-1		
pFliE-mxaF	pDel2-U_*fliE*_-T_*rrnB*_-[P_*mxaF*_-T_*rrnB*_-*Kan*^*R*^]-D_*fliE*_	This study
pFliE-watS1	pFliE-mxaF-U_*fliE*_-T_*rrnB*_ -[P_*mxaF*_-*watS1*-T_*rrnB*_-*Kan*^*R*^]-D_*fliE*_	This study
pFliE-watS2	pFliE-mxaF-U_*fliE*_-T_*rrnB*_ -[P_*mxaF*_-*watS2*-T_*rrnB*_-*Kan*^*R*^]-D_*fliE*_	This study
pFliE-watS3	pFliE-mxaF-U_*fliE*_-T_*rrnB*_ -[P_*mxaF*_-*watS3*-T_*rrnB*_-*Kan*^*R*^]-D_*fliE*_	This study
pFliE-watS4	pFliE-mxaF-U_*fliE*_-T_*rrnB*_ -[P_*mxaF*_-*watS4*-T_*rrnB*_-*Kan*^*R*^]-D_*fliE*_	This study
pFliE-watS5	pFliE-mxaF-U_*fliE*_-T_*rrnB*_ -[P_*mxaF*_-*watS5*-T_*rrnB*_-*Kan*^*R*^]-D_*fliE*_	This study
pFliE-watS1-T7	pFliE-mxaF-U_*fliE*_-T_*rrnB*_ -[P_*mxaF*_-*watS1*-T7-T_*rrnB*_-*Kan*^*R*^]-D_*fliE*_	This study
pFliE-EFTu-watR-Flag	pFliE-mxaF-U_*fliE*_-T_*rrnB*_ -[P_*EFTu*_-*watR-*G4S-Flag-T_*rrnB*_-*Kan*^*R*^]-D_*fliE*_	This study
pWatR-G4S-Flag	pDel2-*watR*-G4S-Flag-[T_*rrnB*_-*Kan*^*R*^]-D_*watR*_	This study
Plasmids for gene expression in *E. coli*		
pGEX-4T-1-WatR	pGEX-4T-1-P_*tac*_-*GST*-*watR*	This study

### Genetic manipulation of *Methylomonas* sp. DH-1

Gene deletion or insertion in *Methylomonas* sp. DH-1 and JHM80 strains were performed as previously described via homologous recombination into the chromosome [4].

### Quantitative reverse transcription PCR (qRT-PCR) and RNA-seq

Total RNA of *Methylomonas* sp. DH-1, JHM15, JHM80, JHM82 and JHM16WF were extracted as previously described with minor modifications [4]. For qRT-PCR analysis, 5 μL of cDNA (diluted 1:200) was amplified by SYBR Green I master mix (Roche-Applied Science, USA) and analyzed with gene-specific primers. The crossing point (Cp) values were processed using Light Cycler 480 software version 1.5 and 2^−ΔΔC^_T_ method was calculated to compare the expression levels of each target genes and normalized by *mxaF* (AYM39_RS15615). Primers for qRT-PCR are listed in Additional file 1: Table S3.

For RNA-seq, two sets of total RNA from *Methylomonas* sp. DH-1 and JHM80 were isolated. 1 μg of total RNA was proceeded to rRNA depletion using NEBNext rRNA depletion kit (Bacteria) (#7850, NEB). Resulted mRNA was used for sequencing library construction by TruSeq Stranded mRNA Library Prep kit (#20020594, Illumina). All experiments were performed following manufacturer's instructions. The prepared sequencing library was sequenced using NovaSeq 6000 (Illumina). The sequencing adapter removal and quality-based trimming on raw data were performed by Trimmomatic (v. 0.36) with default parameter [47]. Cleaned reads were mapped to reference genome (*Methylomonas* sp. DH-1, GCF_001644685.1) using hisat2 (v. 2.2.1) with '-no-spliced-alignment' option [48]. For counting reads which mapped to each CDS, featureCounts in Subread package was used [49]. Finally, normalization of retrieved counts and fold change calculation between groups were performed by DESeq2 package [50].

### ChIP and ChIP-seq analyses

ChIP assay was conducted as previously described with minor modifications using JHM16WF and JHM80WF strains harboring *watR*-Flag [51]. Detailed methods for ChIP are described in Additional file 1: Supplementary Materials and Methods. For ChIP-seq analysis, the eluted DNA after ChIP was extracted with phenol–chloroform-isoamylalcohol (25:24:1) and precipitated with ethanol and glycogen at − 80 °C. 1 ng of prepared DNA was proceeded to sequencing library construction by using NEBNext Ultra II DNA Library Prep Kit for Illumina (#E7645, NEB) following manufacturer's instructions. The sequencing adapter removal and quality-based trimming on raw data were performed by Trimmomatic (v. 0.36) with default parameter [47]. Cleaned reads were mapped to reference genome using bowtie2 (v. 2.4.2) with default parameter. Peak calling was performed by findpeaks command in homer (v. 4.10.3) using "-style factor" parameter [52]. Resulted peaks were annotated by annotatePeaks.pl in homer package. Peaks were transformed to bed file using pos2bed.pl in homer package for detailed analysis. The conserved motifs from peaks were found by MEME-ChIP (v. 4.9.0) [53].

## Supplementary Information

**Additional file 1.** Supplementary Materials and Methods: Western blotting, ChIP analysis, Electrophoretic mobility shift assay (EMSA). **Table S1**. Recipes of the stock solutions of NMS. **Table S2**. Primers used for plasmid and strain construction. **Table S3**. Primers used for qRT-PCR and ChIP-qPCR. **Table S4**. Primers used for EMSA. Table S5. WatR-binding sites determined by ChIP-Seq. **Table S6**. The list of genes induced or repressed upon acetate treatment in the wild-type and △watR strains. **Figure S1**. Amino acid sequence alignment of LTTRs from various species. **Figure S2**. Confirmation of the WatR-Flag strain. **Figure S3**. Confirmation of specific binding of WatR to the probe in EMSA. **Figure S4**. Prediction of transmembrane domains in small peptides encoded from smORFs. **Figure S5**. Increase in acetate tolerance by overexpression of watS1 to watS5. **Figure S6**. Detection of the WatS1 protein.

## Data Availability

The datasets supporting the conclusions of this article are available in the GEO repository. ChIP-seq and RNA-seq data have been deposited in the GEO repository under the accession number of GSE206217 (GSE206215 for RNA-seq and GSE206216 for ChIP-seq).
