# Supplementary material for: Regulation of acetate tolerance by small ORF-encoded polypeptides modulating efflux pump specificity in Methylomonas sp. DH-1
Source: Biotechnol Biofuels Bioprod. 2023 Jul 18;16:114. doi: 10.1186/s13068-023-02364-6 (PMC10355033; doi:10.1186/s13068-023-02364-6)
Supplement: Supplementary file 1 — Additional file 1. Supplementary Materials and Methods: Western blotting, ChIP analysis, Electrophoretic mobility shift assay (EMSA). Table S1. Recipes of the stock solutions of NMS. Table S2. Primers used for plasmid and strain construction. Table S3. Primers used for qRT-PCR and ChIP-qPCR. Table S4. Primers used for EMSA. Table S5. WatR-binding sites determined by ChIP-Seq. Table S6. The list of genes induced or repressed upon acetate treatment in the wild-type and △watR strains. Figure S1. Amino acid sequence alignment of LTTRs from various species. Figure S2. Confirmation of the WatR-Flag strain. Figure S3. Confirmation of specific binding of WatR to the probe in EMSA. Figure S4. Prediction of transmembrane domains in small peptides encoded from smORFs. Figure S5. Increase in acetate tolerance by overexpression of watS1 to watS5. Figure S6. Detection of the WatS1 protein. [file 13068_2023_2364_MOESM1_ESM.docx]

Additional file

**Regulation of acetate tolerance by small ORF-encoded polypeptides modulating efflux pump specificity in *Methylomonas* sp*.* DH-1**

Seungwoo Cha^1^, Yong-Joon Cho^2^, Jong Kwan Lee^1^ and Ji-Sook Hahn^1*^

^1^School of Chemical and Biological Engineering, Institute of Chemical Processes, Seoul National University, 1 Gwanak-ro, Gwanak-gu, Seoul 08826, Republic of Korea

^2^Department of Molecular Bioscience, College of Biomedical Science, Kangwon National University, 1 Gangwondaehakgil, Chuncheon, Gangwon-do 24341, Republic of Korea

^*^Corresponding author: Ji-Sook Hahn

Phone: +82-2-880-9228

Fax: +82-2-888-1604

e-mail: [hahnjs@snu.ac.kr](mailto:hahnjs@snu.ac.kr)

**Supplementary Materials and Methods**

**Western blotting**

Cells were grown in NMS medium with 20% (v/v) methane to OD ~ 1.0 and harvested and washed with IP150 lysis buffer twice. Cell pellets were vortexed with acid-washed glass bead in IP 150 lysis buffer [50 mM Tris-HCl (pH 7.5), 150 mM NaCl, 2 mM MgCl_2_, 0.1% NP40] supplemented with 1 mM PMSF and 0.1% protease inhibitor cocktail (Calbiochem, USA). After boiling with 5x sample buffer [60 mM Tris-HCl (pH 6.8), 0.1% Bromophenol blue, 5% 2-mercaptoethanol, 25% Glycerol, 2% SDS], samples were resolved by 12.5% SDS-PAGE gel and western blotting was performed with anti-DDDDK antibody (MBL life science, USA) and HRP conjugated anti-mouse antibody (Sigma-aldrich, USA), and visualized by G::box Chemi-XL (Syngene, USA).

**ChIP analysis**

Final 2.7% of formaldehyde was added to 50 mL cell culture using 5 mL syringe and crosslinked for 25 min, and quenched with 250 mM glycine for 5 min. After washing once with ice-cold NMS, twice with TBS, and once with lysozyme buffer [(0 mM Tris-HCl (pH 8.0), 20% sucrose, 50 mM NaCl, 10 mM EDTA], cells were resuspended to 500 μL of lysozyme buffer containing 10 mg/mL of lysozyme (Thermo Scientific, USA), 1 mM PMSF and 0.1% protease inhibitor cocktail. After cell lysis with shaking at 37 ^o^C for 30 min, 500 μL of 2x ChIP lysis buffer [100 mM HEPES-KOH (pH 7.5), 300 mM NaCl, 2 mM EDTA, 2% Triton X-100, 0.2% sodium deoxycholate, 0.4% SDS] was added and sonicated 12 times for 20 s (Vitra-cell, Sonics & materials inc, USA) with amplitude 22%. Crude lysates were centrifuged for 20 min and 100 μL of supernatant was used as input. 2 μL of anti-DDDDK antibody was added to proper amounts of lysate (400 μL for JHM80WF strain and 900 μL for JHM16WF strain) and immunoprecipitated overnight at 4^o^C, followed by 2 h incubation with 20 μL Protein A Plus agarose bead (Santa Cruz Biotechnology, USA). After washing the beads, DNA was eluted from beads and treated with RNase and proteinase K. Crosslink was reversed by overnight incubation at 65 ^o^C with 100 mM NaCl, and DNA was purified using Qiagen DNA purification kit. The occupancy of WatR on the target promoter was calculated by dividing the amount of PCR product from the IP sample into input sample, compared to the negative control (*glgA* ORF). The primers used in ChIP-qPCR is listed in Supplementary Table S3.

**Electrophoretic mobility shift assay (EMSA)**

EMSA was performed with biotin-labeled DNA probe, which was prepared via PCR amplification using 5’-biotin modified primer and gel extraction. Total 20 μL of mixture, containing 20 fmole probe and 0.4 to 1.2 μg of GST-WatR protein purified from *E. coli* in binding buffer [100 mM Tris-HCl (pH 7.5), 500 mM KCl, 10 mM DTT, 60% glycerol, 5 mM EDTA, 500 μg/mL BSA, 500 μg/mL salmon sperm DNA] was incubated at room temperature for 20 min. After adding 5x sample buffer without SDS [60 mM Tris-HCl (pH 6.8), 0.1% bromophenol blue, 5% 2-mercaptoethanol, 25% glycerol], the mixture was resolved via electrophoresis in 6% native polyacrylamide gel with 0.5X TBE buffer [40 mM Tris base, 45 mM Boric acid, and 1 mM EDTA]. DNA was transferred to hybond-N^+^ membrane (GE healthcare Amersham, USA), and crosslinked for 10 min using UV lamp (Korea Ace Sci., Korea) device under 254 nm wavelength. The membrane was incubated with HRP-conjugated streptavidin (Thermofisher scientific, USA) and the signals were visualized by G::box Chemi-XL (Syngene, USA). The primers for EMSA and competition assay are listed in Supplementary Table S4.

**Table S1** Recipes of the stock solutions of NMS

| Ingredient | Amount |
| --- | --- |
| 1000X trace element solution | |
| FeSO_4_·7H_2_O | 500 mg/L |
| ZnSO_4_·7H_2_O | 400 mg/L |
| MnCl_2_·7H_2_O | 20 mg/L |
| CoCl_2_·6H_2_O | 50 mg/L |
| NiCl_2_·6H_2_O | 10 mg/L |
| H_3_BO_3_ | 15 mg/L |
| EDTA | 250 mg/L |
| 100X vitamin stock | |
| Biotin | 2.0 mg/L |
| Folic acid | 2.0 mg/L |
| Thiamine HCl | 5.0 mg/L |
| Ca pantothenate | 5.0 mg/L |
| Vitamin B12 | 0.1 mg/L |
| Riboflavin | 5.0 mg/L |
| Nicotinamide | 5.0 mg/L |
| 100X phosphate stock solution | |
| KH_2_PO_4_ | 26 g/L |
| Na_2_HPO_4_ | 32.8 g/L |

**Table S2** Primers used for plasmid and strain construction

| Forward primer (5’ to 3’)* | | Reverse primer (5’ to 3’)* | | Usage | |
| --- | --- | --- | --- | --- | --- |
| Primers used for gene deletion in *Methylomonas* sp. DH-1 | | | | |  |
| actGCGGCCGCTCACCAGCCTGTTAGGTG | | actACTAGTATATTGTCGGCGAAATTCGT | | Cloning U*_watO_* of pDel2-watABPO(K) with *Not*I/*Spe*I |  |
| actGGGCCCTCGCTTCGGCTAACAGTG | | actGAGCTCCCCAACAAAGCGATAGCG | | Cloning D*_watA_* of pDel2-watABPO(K) with *Apa*I/*Sac*I |  |
| gcgATTTAAATCGAATTGGACAGTCCCGCCAC | | gcgACTAGTGGTCTACTCCAAAAGTTGAACACATGGC | | Cloning U*_watS1_* of pDel2-watS1 with *Swa*I/*Spe*I |  |
| actGGGCCCCCGCTTGCGCCCGGCGCGGG | | gcgGAGCTCGCCGTCAAGCGGGCTGCACTTTATC | | Cloning D*_watS1_* of pDel2-watS1 with *Apa*I/*Sac*I |  |
| actGGGCCCGCGGAACCCCTATTTGT | | gcgTTAATTAATCAAGAAGATCCTTTGATC | | Cloning *Amp^R^* of pDel2-watABPO(A) with *Apa*I/*Pac*I |  |
| Primers used for gene expression in *Methylomonas* sp. DH-1 | | | | |  |
| gactGCGGCCGCTAGCGAAAACCAACGTGACG | | actCGCGCGCGGATATTGACTCGTGTGTATTGCCTGCC | | Cloning U*_fliE_* of pFliE-mxaF with *Not*I/*Mau*BI |  |
| tataGGGCCCGTAGCGGAAGCCGGCCA | | tataGGCGCGCCGGCACGCCTTGAACTT | | Cloning D*_fliE_* of pFliE-mxaF with *Apa*I/*Asc*I |  |
| actGGCGCGCCCAAATAAAACGAAAGGCTCAGTCGG | | actCGCGCGCGATTTGTCCTACTCAGGAGAGCGTTCACCG | | Cloning T*_rrnB_* of pFliE-mxaF with *Asc*I/*Mau*BI |  |
| gggCGCGCGCGCTGATTTTGTTTGCCACAGGC | | gcgGGATCCGAATCCTCCTAAGTTGTTTATTAGAGTGC | | Cloning P*_mxaF_* of pFliE-mxaF with *Mau*BI/*Bam*HI |  |
| gcgGGATCCATGACTAACGTACAAAAAGACATTCTGAACG | | gcgACTAGTTCAGTCTTTTTGATTTTTCCGGTTTAGGTTG | | Cloning *watS1* of pFliE-watS1 with *Bam*HI/*spe*I |  |
| actGGATCCATGAGCAAATCTAACAATCTTTATCGAG | | gcgACTAGTTTAAGCGCGGAACGAGCC | | Cloning *watS2* of pFliE-watS2 with *Bam*HI/*spe*I |  |
| gcgGGATCCATGAACCAGTCAAAATTTAAAGACATCG | | gcgACTAGTTTAAATCCGGACCGGTTTGGCAAC | | Cloning *watS3* of pFliE-watS3 with *Bam*HI/*spe*I |  |
| gcgGGATCCATGAATACCCCGACGTTCTATCG | | gcgACTAGTTCAGATGCGTACCGGTTTGG | | Cloning *watS4* of pFliE-watS4 with *Bam*HI/*spe*I |  |
| gcgGGATCCATGGAACTTGTTATCCAACCGGTTC | | gcgACTAGTTCAGGCAGCGATAGAGGCTGTAGAG | | Cloning *watS5* of pFliE-watS5 with *Bam*HI/*spe*I |  |
| gcgGCGGCCGCGCAACTTCCAACGCCACCGG | | gcgACTAGTTCACTTGTCATCGTCATCCTTGTAATCagaaccacccccgccTGGGTTCATGCCGATCCG | | Cloning *watR*-G4S-Flag of pWatR-G4S-Flag with *Not*I/*Spe*I |  |
| gcgGGGCCCGCGAACTGCAACGCAAGTGGG | | gcgGAGCTCGGCCGGCGCGGAAGTGGGCTGG | | Cloning *watR* downstream 1 kb to pWatR-G4S-Flag with *Apa*I/*Sac*I |  |
| Primers used for gene expression in *E. coli* | | | | |  |
| gcgGAATTCATGGACAAACTAACCAGCATGAACG | | gcgGCGGCCGCTCATGGGTTCATGCCGATCC | | Cloning *watR* to pGEX-4T-1 vector with *Eco*RI/*Not*I |  |

* Underlined upper case sequences indicate restriction enzyme sites. Underlined lower case sequences indicate G4S linker sequence

**Table S3** Primers used for qRT-PCR and ChIP-qPCR

| Forward primer (5’ to 3’) | Reverse primer (5’ to 3’) | Target gene  or locus | Gene number  (AYM39_RS) |
| --- | --- | --- | --- |
| Primers for qRT-PCR | | | |
| TGGAAGGCAAACAGGCCAAT | gcaagcggtactctatgctcttgt | *glgA* | 03775 |
| TATCTGGAACGCTGCCAGCA | TGACCGCCTTTCAGCACCAT | *watR* | 21130 |
| GCCTATGCCGACACCAAAAC | GTGGTTGTACGATTCCTGGGTGG | *watO* | 17405 |
| CGATTGCAACCCAGCCCAAAGCG | GCGGACCAATTGCCACATCAGCAG | *watP* | 17395 |
| AATATTGTTTCGTGCGGGCGcc | CAGAAACACTTCGGCCTCCTgg | *watA* | 17390 |
| GGCCATAGCCGGTTTTGGTTCGGT | GGGTTACTGCAAACAAGCGGGCC | *watB* | 17385 |
| gcgGGATCCATGACTAACGTACAAAAAGACATTCTGAACG | gcgACTAGTTCAGTCTTTTTGATTTTTCCGGTTTAGGTTG | *watS1* | (unannotated) |
| TTGTGTCGCGGCCGGCATCGCCTGCCTGTGG | GGCTTTGGCCACATACTCGCCGATGCGCG | *gltA1* | 19040 |
| Primers for ChIP-qPCR | | | |
| GTTTGCCAGCCACGCGTTGCGT | CTATTCTAACCCCGATCGGACAAGC | *watP* promoter | 17395 |
| GGCGGCCTGGGGCAAGGTCG | CTCGGCTTCCAGTTCGAAACGGC | *bioD* promoter | 08175 |
| GCCGCAGTAAACATTCCGTCAATCGC | GGCGGCCGCGCCGGCCGCGTCACCC | *pmmM* promoter | 04660 |
| TAAATTCATTCATTGTGCTCAAATCC | CGTTGACATTTTTATTGTTTCTTAATTTCATACAA | *watR* promoter | 21130 |
| GCCACCGCAGCGAGATCAGG | gcgACTAGTGGTCTACTCCAAAAGTTGAACACATGGC | *watS1* promoter | (unannotated) |

**Table S4** Primers used for EMSA

| Primer (5’ to 3’)* | Description |
| --- | --- |
| CTTGGAAGGAGGTTACCGCTCG | Forward primer for the 590-, and 588-bp P*_watR_* and P*_watRΔTT_* probes (Figure 2C) |
| CTCGCCGGCTTCAGTCAGGCTTAAG | Reverse primer for P*_watR_* competition assay (Figure S3) |
| Biot-CTCGCCGGCTTCAGTCAGGCTTAAG | Reverse primer for the 590- and 588-bp P*_watR_* and P*_watRΔTT_* probes (Figure 2C) |

* Biot indicates biotin label.

**Table S5** WatR-binding sites determined by ChIP-Seq

| The nearest gene AMR39_RS | Promoter region | Name | Function | Reannotated gene |
| --- | --- | --- | --- | --- |
| 00605 | + |  |  | *watS1* |
| 02245 | *-* |  |  | *watS2* |
| 07485 | + |  |  | *watS3* |
| 19610 | + |  |  | *watS4* |
| 13560 | *-* |  |  | *watS5* |
| 17395 | + | *watP* | hypothetical protein |  |
| 00705 | + |  | FtsH/Yme1/Tma family protein |  |
| 02410 | + |  | hypothetical protein |  |
| 02950 | + |  | hypothetical protein |  |
| 04660 | + | *pmmM* | phosphomannomutase |  |
| 06470 | + |  | CRISPR_1 |  |
| 06860 | + |  | hypothetical protein |  |
| 08175 | + | *bioD* | dethiobiotin synthase |  |
| 09830 | + |  | sulfate ABC transporter |  |
| 11160 | + |  | hypothetical protein |  |
| 15960 | + |  | hypothetical protein |  |
| 19040 | + | *gltA1* | citrate-synthase |  |
| 20750 | + |  | hypothetical protein |  |
| 02830 | - |  | methionine adenosyltransferase |  |
| 06815 | - |  | GDP-L-fucose synthase |  |
| 07170 | - |  | aspartate kinase |  |
| 19890 | *-* |  | S41 family peptidase |  |

**Table S6.** The list of genes induced or repressed upon acetate treatment in the wild-type and *△watR* strains

| WatR-dependent acetate induction | | |  | | |  | |  | |  | |  | |  |
| --- | --- | --- | --- | --- | --- | --- | --- | --- | --- | --- | --- | --- | --- | --- |
|  |  |  |  | | |  | |  | |  | |  | |  |
| gene number AYM39_RS | function | SmORF | log2(acetate/control) | | | | | log2(Fc WT/Fc ΔwatR) | | log2(Fc WT/Fc ΔwatR) >1 | | WatR-dependent induction (manual curation) | | Binding of WatR |
|  |  |  | WT | | | *ΔwatR* | |  |  |  |  |  |  |  |
| 00605 | hypothetical protein |  | | 1.126 | -0.073 | | 1.199 | | O | | O | | O | |
|  |  | *watS1* | | 1.911 | 0.001 | | 1.910 | |  | | O | | O | |
|  |  | *watS2* | | 3.232 | 0.122 | | 3.110 | |  | | O | | O | |
|  |  | *watS3* | | 1.703 | 0.029 | | 1.674 | |  | | O | | O | |
|  |  | *watS4* | | 2.279 | 0.333 | | 1.946 | |  | | O | | O | |
| 13560 | glutathione S-transferase |  | | 2.986 | -0.037 | | 3.023 | | O | |  | |  | |
|  |  | *watS5* | | 4.541 | 0.170 | | 4.371 | |  | | O | | O | |
| 17390 | subunit of RND-type efflux pump (watA) |  | | 1.426 | 0.296 | | 1.130 | | O | | O | | O | |
| 17385 | subunit of RND-type efflux pump (watB) |  | | 1.218 | 0.255 | | 0.963 | |  | | O | | O | |
| 17395 | hypothetical protein (watP) |  | | 1.078 | 0.340 | | 0.738 | |  | | O | | O | |
| 08535 | hypothetical protein |  | | 3.681 | -0.197 | | 3.878 | | O | | O | |  | |
| 07495 | glutathione S-transferase |  | | 1.022 | -0.265 | | 1.287 | | O | | O | |  | |
| 08545 | ATP-grasp domain-containing protein |  | | 1.014 | -0.077 | | 1.091 | | O | | O | |  | |
|  |  |  |  | | |  | |  | |  | |  | |  |
| WatR-independent acetate induction | | |  | | |  | |  | |  | |  | |  |
|  |  |  |  | | |  | |  | |  | |  | |  |
| gene number AYM39_RS | function |  | log2(acetate/control) | | | | | log2(Fc WT/Fc ΔwatR) | |  | |  | |  |
|  |  |  | WT | | | *ΔwatR* | |  |  |  | |  | |  |
| 09160 | hypothetical protein |  | 3.105 | | | 2.553 | | 0.552 | |  | |  | |  |
| 17295 | MCE family protein |  | 2.829 | | | 2.998 | | -0.169 | |  | |  | |  |
| 17305 | CHAT domain-containing protein |  | 2.684 | | | 2.502 | | 0.182 | |  | |  | |  |
| 17300 | caspase family protein |  | 2.636 | | | 2.791 | | -0.155 | |  | |  | |  |
| 09155 | peptidase domain-containing ABC transporter |  | 2.577 | | | 2.426 | | 0.151 | |  | |  | |  |
| 17290 | lysozyme |  | 2.493 | | | 2.645 | | -0.152 | |  | |  | |  |
| 11750 | hybrid non-ribosomal peptide synthetase/type I polyketide synthase |  | 2.416 | | | 2.228 | | 0.188 | |  | |  | |  |
| 09145 | hypothetical protein |  | 2.358 | | | 2.860 | | -0.502 | |  | |  | |  |
| 09135 | hypothetical protein |  | 2.354 | | | 2.256 | | 0.098 | |  | |  | |  |
| 09140 | hypothetical protein |  | 2.354 | | | 2.599 | | -0.245 | |  | |  | |  |
| 09150 | HlyD family efflux transporter periplasmic adaptor subunit |  | 2.346 | | | 2.250 | | 0.096 | |  | |  | |  |
| 08110 | hypothetical protein |  | 2.165 | | | 2.244 | | -0.079 | |  | |  | |  |
| 17310 | hypothetical protein |  | 1.981 | | | 1.833 | | 0.148 | |  | |  | |  |
| 09165 | cell envelope integrity protein CreD |  | 1.969 | | | 1.732 | | 0.237 | |  | |  | |  |
| 20175 | hypothetical protein |  | 1.849 | | | 1.967 | | -0.118 | |  | |  | |  |
| 17320 | phage tail sheath family protein |  | 1.835 | | | 2.409 | | -0.574 | |  | |  | |  |
| 11805 | hypothetical protein |  | 1.8 | | | 1.952 | | -0.152 | |  | |  | |  |
| 06155 | squalene/phytoene synthase family protein |  | 1.76 | | | 1.698 | | 0.062 | |  | |  | |  |
| 17285 | autotransporter outer membrane beta-barrel domain-containing protein |  | 1.697 | | | 1.788 | | -0.091 | |  | |  | |  |
| 18680 | sulfate adenylyltransferase |  | 1.675 | | | 3.117 | | -1.442 | |  | |  | |  |
| 11745 | non-ribosomal peptide synthetase |  | 1.637 | | | 1.869 | | -0.232 | |  | |  | |  |
| 20170 | hypothetical protein |  | 1.617 | | | 1.912 | | -0.295 | |  | |  | |  |
| 04975 | diguanylate cyclase |  | 1.556 | | | 1.878 | | -0.322 | |  | |  | |  |
| 18685 | sulfate adenylyltransferase subunit CysD |  | 1.517 | | | 1.896 | | -0.379 | |  | |  | |  |
| 06150 | squalene--hopene cyclase |  | 1.501 | | | 1.631 | | -0.130 | |  | |  | |  |
| 06145 | phosphorylase |  | 1.42 | | | 1.639 | | -0.219 | |  | |  | |  |
| 11800 | hypothetical protein |  | 1.33 | | | 1.585 | | -0.255 | |  | |  | |  |
| 13875 | hypothetical protein |  | 1.312 | | | 1.156 | | 0.156 | |  | |  | |  |
| 11810 | hypothetical protein |  | 1.234 | | | 1.169 | | 0.065 | |  | |  | |  |
| 18675 | YchE family NAAT transporter |  | 1.217 | | | 2.835 | | -1.618 | |  | |  | |  |
| 23920 | hypothetical protein |  | 1.198 | | | 0.749 | | 0.449 | |  | |  | |  |
| 05670 | hypothetical protein |  | 1.165 | | | 1.327 | | -0.162 | |  | |  | |  |
| 18670 | hypothetical protein |  | 1.162 | | | 2.721 | | -1.559 | |  | |  | |  |
| 17085 | hypothetical protein |  | 1.127 | | | 0.765 | | 0.362 | |  | |  | |  |
| 06140 | aspartate aminotransferase family protein |  | 1.089 | | | 0.945 | | 0.144 | |  | |  | |  |
| 20150 | HAD-IC family P-type ATPase |  | 1.074 | | | 1.200 | | -0.126 | |  | |  | |  |
| 15345 | EamA family transporter |  | 1.071 | | | 1.520 | | -0.449 | |  | |  | |  |
| 17080 | hypothetical protein |  | 1.07 | | | 0.929 | | 0.141 | |  | |  | |  |
| 12350 | ABC transporter ATP-binding protein |  | 1.059 | | | 1.532 | | -0.473 | |  | |  | |  |
| 12345 | phosphoenolpyruvate carboxylase |  | 1.024 | | | 1.645 | | -0.621 | |  | |  | |  |
| 06135 | copper resistance protein CopC |  | 1.006 | | | 1.009 | | -0.003 | |  | |  | |  |
|  |  |  |  | | |  | |  | |  | |  | |  |
| Acetate repression | |  |  | | |  | |  | |  | |  | |  |
|  |  |  |  | | |  | |  | |  | |  | |  |
| gene number AYM39_RS | function |  | log2(acetate/control) | | | | | log2(Fc WT/Fc ΔwatR) | | WatR-dependent repression (RNA-seq) | |  | |  |
|  |  |  | WT | | | *ΔwatR* | |  |  |  |  |  | |  |
| 18695 | NADH dehydrogenase |  | -2.713 | | | -0.067 | | -2.646 | | O | |  | |  |
| 18690 | DUF2309 domain-containing protein |  | -1.345 | | | 0.649 | | -1.994 | | O | |  | |  |
| 03490 | chaperonin GroEL |  | -2.7 | | | -2.446 | | -0.254 | |  | |  | |  |
| 17600 | RNA-binding protein |  | -2.634 | | | -3.664 | | 1.030 | |  | |  | |  |
| 22490 | ThiF family adenylyltransferase |  | -2.458 | | | -1.946 | | -0.512 | |  | |  | |  |
| 15300 | molecular chaperone HtpG |  | -2.423 | | | -2.558 | | 0.135 | |  | |  | |  |
| 03485 | co-chaperone GroES |  | -2.319 | | | -2.443 | | 0.124 | |  | |  | |  |
| 22735 | DUF1508 domain-containing protein |  | -2.169 | | | -1.988 | | -0.181 | |  | |  | |  |
| 23475 | hypothetical protein |  | -2.124 | | | -1.978 | | -0.146 | |  | |  | |  |
| 14200 | molecular chaperone DnaK |  | -1.858 | | | -1.669 | | -0.189 | |  | |  | |  |
| 17605 | cold-shock protein |  | -1.784 | | | -2.422 | | 0.638 | |  | |  | |  |
| 14205 | nucleotide exchange factor GrpE |  | -1.761 | | | -1.676 | | -0.085 | |  | |  | |  |
| 23170 | type II toxin-antitoxin system RelE/ParE family toxin |  | -1.687 | | | -1.593 | | -0.094 | |  | |  | |  |
| 14185 | molecular chaperone DnaJ |  | -1.634 | | | -1.554 | | -0.080 | |  | |  | |  |
| 14195 | hypothetical protein |  | -1.617 | | | -1.662 | | 0.045 | |  | |  | |  |
| 14180 | 4-hydroxy-tetrahydrodipicolinate reductase |  | -1.591 | | | -1.406 | | -0.185 | |  | |  | |  |
| 14190 | hypothetical protein |  | -1.482 | | | -1.667 | | 0.185 | |  | |  | |  |
| 17595 | DEAD/DEAH box helicase |  | -1.44 | | | -2.657 | | 1.217 | |  | |  | |  |
| 15020 | ribonuclease HII |  | -1.248 | | | -0.963 | | -0.285 | |  | |  | |  |
| 12585 | RNA polymerase sigma factor RpoH |  | -1.152 | | | -0.845 | | -0.307 | |  | |  | |  |
| 14210 | heat-inducible transcriptional repressor HrcA |  | -1.123 | | | -0.949 | | -0.174 | |  | |  | |  |
| 13175 | ADP-glyceromanno-heptose 6-epimerase |  | -1.086 | | | -1.231 | | 0.145 | |  | |  | |  |
| 16265 | lipopolysaccharide heptosyltransferase I |  | -1.033 | | | -0.865 | | -0.168 | |  | |  | |  |
|  |  |  |  | | |  | |  | |  | |  | |  |

**
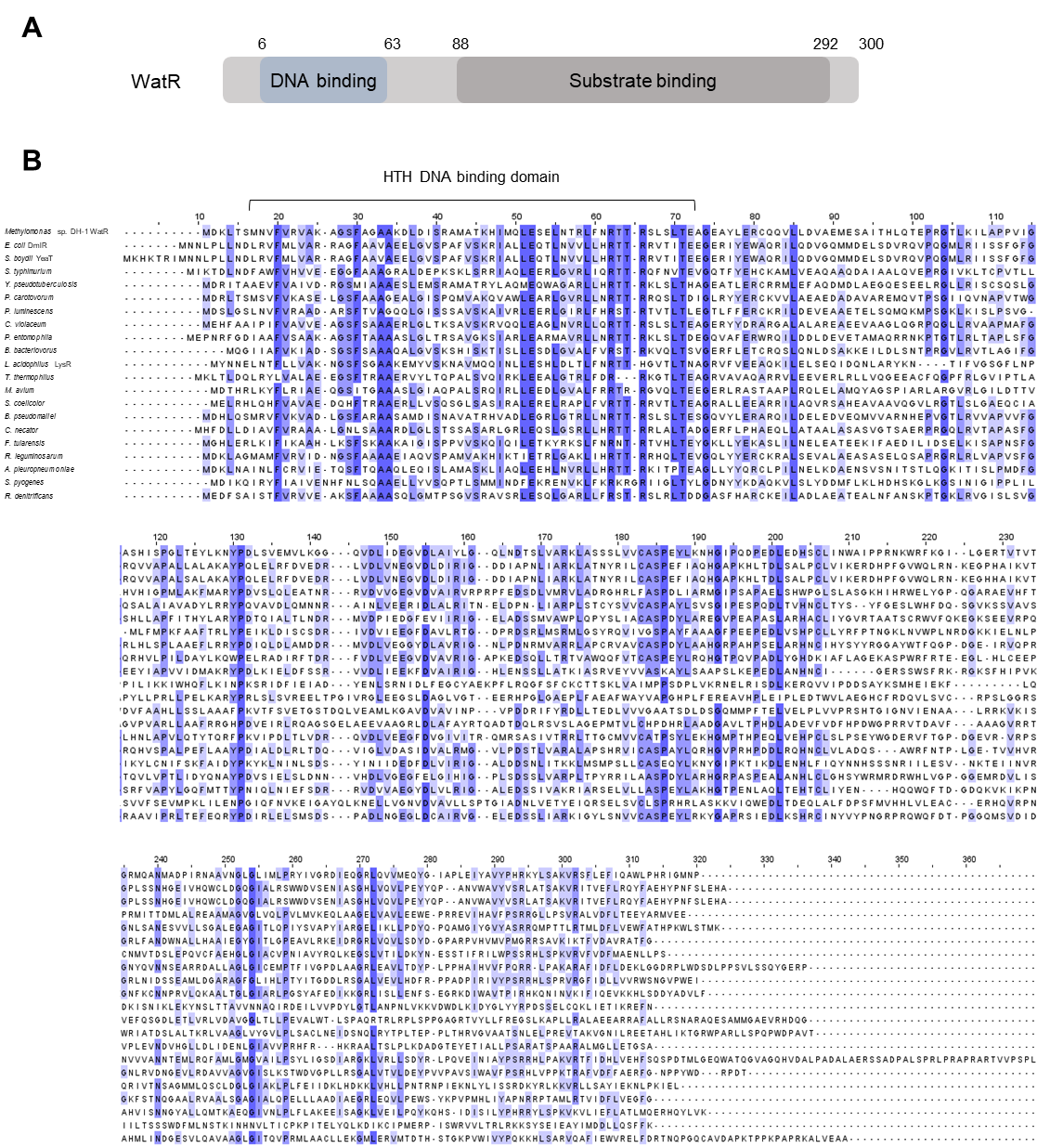
**

**Figure S1** Amino acid sequence alignment of LTTRs from various species.

(A) Domain structures of WatR.

(B) LTTRs from several bacterial species that show high homology with WatR of DH-1 were aligned using Jalview 2.11.2.3. The highly conserved helix turn helix (HTH) DNA binding domain is marked.

**
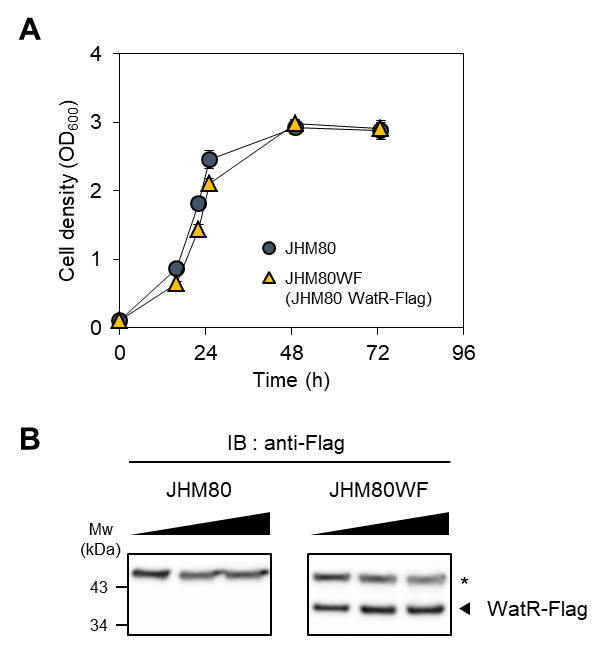
**

**Figure S2** Confirmation of the WatR-Flag strain.

(A) Growth curve for JHM80 and JHM80WF (JHM80 WatR-Flag) strains used for ChIP and ChIP-seq. Three independent experiments were performed, and error bars indicate standard deviations.

(B) Expression test for the Flag-tagged WatR protein, with its control JHM80 strain. 50, 75, 100 μg of whole cell lysates were loaded and analyzed by immune blotting (IB). The asterisk indicates a non-specific band.

**
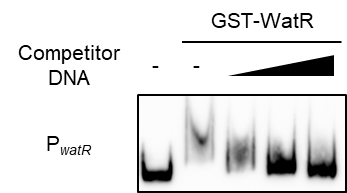
**

**Figure S3** Confirmation of specific binding of WatR to the probe in EMSA.

1.2 μg of WatR protein and 20 fmole of biotin-labeled P*_watR_* probe were used for EMSA. The competition assay for the WatR binding was conducted with 10X, 25X, 50X excess amount of unlabeled probe.

**
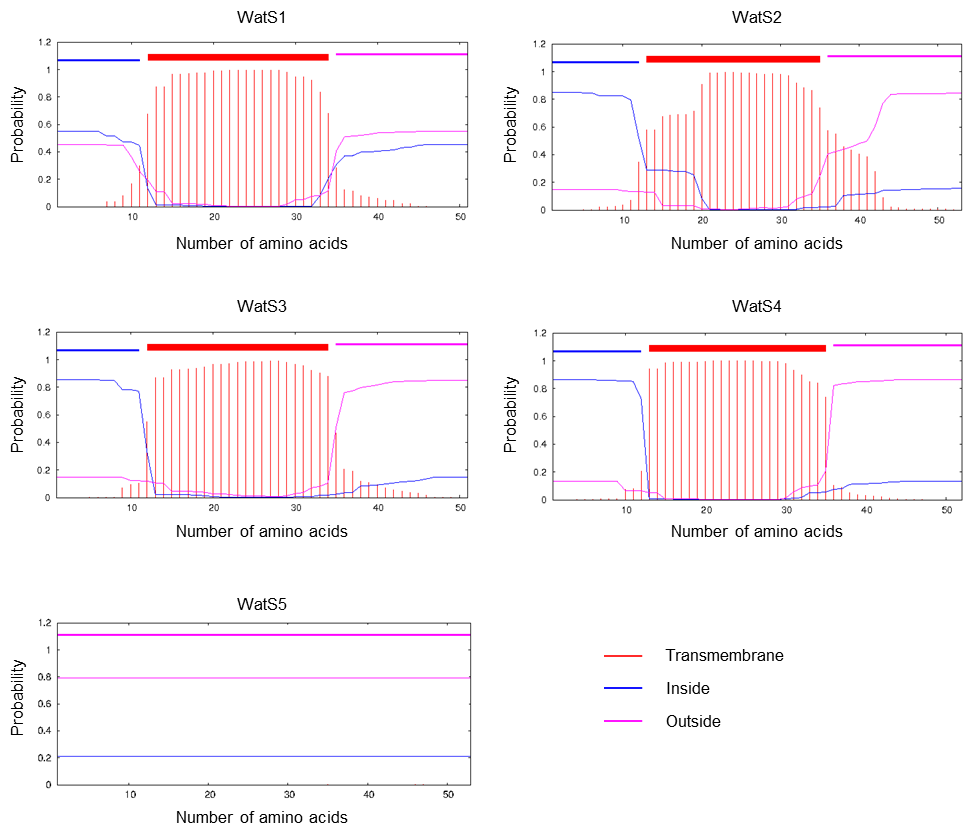
**

**Figure S4** Prediction of transmembrane domains in small peptides encoded from smORFs.

The putative membrane domains of WatS1 to WatS5 were analyzed by TMHMM 2.0.

**
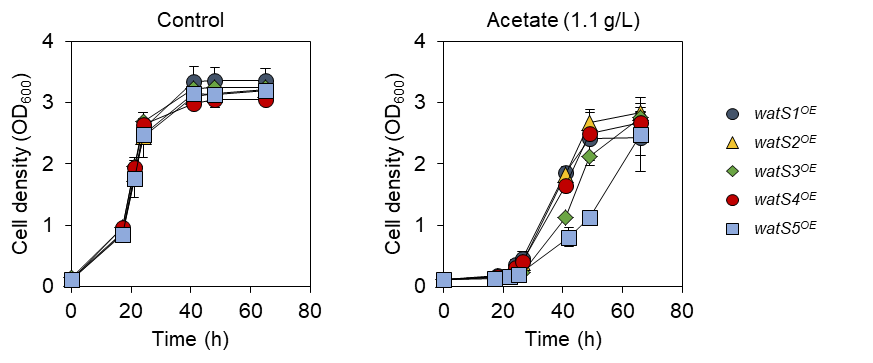
**

**Figure S5** Increase in acetate tolerance by overexpression of *watS1* to *watS5*.

All strains were grown in NMS media with 20% (v/v) methane with or without 1.1 g/L acetate. Each value represents the average ± standard deviations from two independent experiments.

**
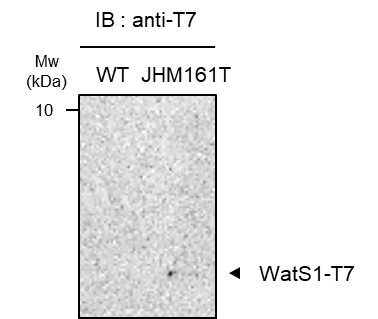
**

**Figure S6** Detection of the WatS1 protein.

Expression test for the T7 epitope tagged WatS1 protein, with its control DH-1 strain. 50 μg of whole cell lysates of wild type and JHM161T strain harboring *watS1-T7* were analyzed by immune blotting with anti-T7 antibody. The WatS1-T7 band is indicated.

|  |  |  |  |  |  |  |  |  |
| --- | --- | --- | --- | --- | --- | --- | --- | --- |

**References**

1. Marx CJ, Lidstrom ME. 2002. Broad-host-range cre-lox system for antibiotic marker recycling in gram-negative bacteria. Biotechniques 33:1062-7.

2. Henard CA, Smith H, Dowe N, Kalyuzhnaya MG, Pienkos PT, Guarnieri MT. 2016. Bioconversion of methane to lactate by an obligate methanotrophic bacterium. Sci Rep 6:21585.

3. Bolger AM, Lohse M, Usadel B. 2014. Trimmomatic: a flexible trimmer for Illumina sequence data. Bioinformatics 30:2114-2120.

4. Kim D, Paggi JM, Park C, Bennett C, Salzberg SL. 2019. Graph-based genome alignment and genotyping with HISAT2 and HISAT-genotype. Nat Biotechnol 37:907-915.

5. Liao Y, Smyth GK, Shi W. 2013. The Subread aligner: fast, accurate and scalable read mapping by seed-and-vote. Nucleic Acids Res 41:e108.

6. Love MI, Huber W, Anders S. 2014. Moderated estimation of fold change and dispersion for RNA-seq data with DESeq2. Genome Biol 15:550.

7. Lee JK, Kim S, Kim W, Kim S, Cha S, Moon H, Hur DH, Kim SY, Na JG, Lee JW, Lee EY, Hahn JS. 2019. Efficient production of d-lactate from methane in a lactate-tolerant strain of Methylomonas sp. DH-1 generated by adaptive laboratory evolution. Biotechnol Biofuels 12:234.
